# A genome-wide association study identifies an African-specific locus on chromosome 21q22.12 associated with Burkitt lymphoma risk and survival

**DOI:** 10.1038/s41375-025-02690-8

**Published:** 2025-07-11

**Authors:** Diptavo Dutta, Mateus H. Gouveia, Bryan R. Gorman, Atuahene Adu-Gyamfi, Chia-Han Lee, Martin D. Ogwang, Patrick Kerchan, Steven J. Reynolds, Constance N. Tenge, Pamela A. Were, Walter N. Wekesa, Robert K. Tenge, Nestory Masalu, Esther L. Kawira, Tobias Kinyera, Isaac Otim, Hadijah Nabalende, Herry Dhudha, Bosco Candia, Janet Abaru, Wusheng Yan, Oscar Florez-Vargas, Yi Xie, Michelle Ho, Leona W. Ayers, Kishor Bhatia, James J. Goedert, Ruth M. Pfeiffer, Michelle Manning, Amy Hutchinson, Nathan Cole, Wen Luo, Belynda Hicks, George Chagaluka, W. Thomas Johnston, Nora Mutalima, Eric Borgstein, George N. Liomba, Steven Kamiza, Nyengo Mkandawire, Elizabeth M. Molyneux, Collins Mitambo, Robert Newton, Reiner Siebert, Michael Dean, Meredith Yeager, Stephen J. Chanock, Ludmila Prokunina-Olsson, Sam M. Mbulaiteye

**Affiliations:** 1 Integrative Tumor Epidemiology Branch, Division of Cancer Epidemiology & Genetics, National Cancer Institute, Bethesda, MD USA; 2 Center for Research on Genomics & Global Health, Division of Intramural Research, National Human Genome Research Institute, Bethesda, MD USA; 3 Trans-Divisional Research Program, Division of Cancer Epidemiology & Genetics, National Cancer Institute, Bethesda, MD USA; 4 Division of Cancer Epidemiology & Genetics, Laboratory of Translational Genomics, National Cancer Institute, Bethesda, MD USA; 5 Department of Surgery, St. Mary’s Hospital, Lacor, Gulu Uganda; 6 Medical Superintendent’s Office, Kuluva Hospital, Arua, Uganda; 7 Division of Intramural Research, National Institute of Allergy and Infectious Diseases, Bethesda, MD USA; 8 Department of Pediatrics, Moi University, Eldoret, Kenya; 9 EMBLEM Study, Academic Model Providing Access To Healthcare (AMPATH), Moi Teaching and Referral Hospital, Eldoret, Kenya; 10 Department of Pathology, Moi University, Eldoret, Kenya; 11 Department of Surgery, Moi University, Eldoret, Kenya; 12 Department of Oncology, Bugando Medical Center, Mwanza, Tanzania; 13 EMBLEM Study, Shirati Health, Education, and Development Foundation, Shirati, Tanzania; 14 EMBLEM Study, African Field Epidemiology Network, Gulu, Uganda; 15 Department of Pathology, Ohio State University, Columbus, OH USA; 16 Infections and Immunoepidemiology Branch, Division of Cancer Epidemiology & Genetics, National Cancer Institute, Bethesda, MD USA; 17 Biostatistics Branch, Division of Cancer Epidemiology & Genetics, National Cancer Institute, Bethesda, MD USA; 18 Division of Cancer Epidemiology & Genetics, Cancer Genomics Research Laboratory, National Cancer Institute, Bethesda, MD USA; 19 Departments of Pediatrics and Surgery, University of Malawi, Blantyre, Malawi; 20 Department of Health Sciences, Epidemiology and Cancer Statistics Group, University of York, York, UK; 21 Cancer Epidemiology Unit, University of Oxford, Oxford, UK; 22 Research Department, National Health Sciences Research Committee, Ministry of Health, Lilongwe, Malawi; 23 Institute of Human Genetics, Ulm University and Ulm University Medical Center, Ulm, Germany; 24 German Center for Child and Adolescent Health (DZKJ), Ulm, Germany; 25 Division of Cancer Epidemiology & Genetics, National Cancer Institute, Bethesda, MD USA

**Keywords:** Genetics research, Cancer epidemiology

## Abstract

Burkitt lymphoma (BL) is a B-cell malignancy that disproportionately affects children in sub-Saharan Africa. We performed a genome-wide association study (GWAS) in a combined set of 800 childhood cases and 3865 controls in East Africa, controlling for age, sex, country, population-specific principal components, and a genetic relationship matrix. This analysis identified a BL-protective region within chromosome 21q22.12 tagged by the rs111457485-T allele (odds ratio [OR] = 0.57; *p* = 5.7 × 10^−9^). The results were robust in standard meta-analysis (OR = 0.57, *p* < 1.6 × 10^−8^), sensitivity analyses (removing genomic outliers and related individuals), and after adjustment for Epstein-Barr virus (EBV) status. Genomic analyses revealed long-range (over ~700 kb) chromatin interactions between the chr21q22.12 locus and the *RUNX1*-P1 promoter region. The African-specific rs2242780-C allele (*r*^*2*^ = 0.69 with the rs111457485-T allele in the study controls) showed increased enhancer activity in in-vitro Luciferase reporter assays (*p* = 4.5 × 10^−10^), nominating it as the likely functional variant for the BL-associated loci. In addition to the association with reduced BL risk in GWAS (OR = 0.62, *p* = 2.24 × 10^−8^), the rs2242780-C allele was also associated with better survival in patients with abdominal-only BL in exploratory analyses (hazard ratio = 0.39, *p* = 0.038, 106 patients, 59 deaths). Our GWAS uncovered novel BL-protective loci near *RUNX1*, offering insights into the genetic etiology of BL in African children.

## Introduction

Burkitt lymphoma (BL) is a germinal center (GC) B-cell malignancy that accounts for most childhood cancers in many countries in sub-Saharan Africa (SSA) [1] and ~30% of specified childhood lymphoid malignancies in most developed countries [2]. The elevated BL risk in SSA (10–20-fold higher than that elsewhere [3]) is attributed to Epstein–Barr virus (EBV) [4], a Class 1 carcinogen [1, 5], and *Plasmodium falciparum* [6–8], the parasite that causes malaria and is classified as a Class 2A carcinogen. EBV and *P. falciparum* influence the risk of *IG*::*MYC* translocations, which are a hallmark feature of BL [1], by upregulating activation-induced cytidine deaminase (AICDA) [9], a mutator enzyme involved in class switch recombination and somatic hypermutation in B-cells [10, 11]. Additional somatic [1] or epigenetic [12] changes acquired in driver genes such as *TP53*, *ID3*, *TCF3*, and *SMARCA4* [1, 13–15] enable progression to BL [1].

**Fig. 1:**
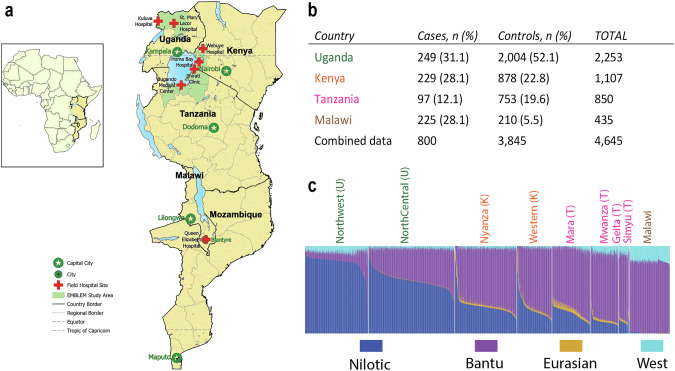
BL GWAS sites, sample sizes, and ancestral admixture patterns. **a** Map showing the countries and geographic locations where BL patients and controls contributing to the GWAS were enrolled; green shading indicates geographical areas where participants in EMBLEM were recruited in Uganda, Tanzania, and Kenya. The red crosses mark the locations of the hospitals where BL patients were diagnosed, enrolled, and treated. The stars on a green background mark the capital cities in each country where the tertiary-level cancer care centers are located and are generally difficult to reach for poor patients in the rural study areas. In Malawi, cases and controls were enrolled at a tertiary cancer care hospital in Blantyre without restrictions on geographical areas of origin. The map was drawn via ESRI ArcGIS Pro software. No portions of this figure were imported as image components from a database. **b** Sample size in the BL GWAS (*n* = 4645) by case status and country. **c** Ancestry admixture plots of participants in the BL GWAS, by enrollment region and country. The font colors of the regions are matched to the font colors of the countries in the table. The ancestral admixture plots are presented with the regions sorted in a southeastward direction to highlight gradients of the dominant Nilotic and Bantu ancestries in the northern and southern regions, respectively. The proportion of Nilotic ancestry is highest in the northcentral region and lowest in Malawi, whereas that of Bantu ancestry is lowest in the northcentral region and highest in Malawi. The West African and Eurasian ancestries make minor contributions in some of the regions. In the BL GWAS, the population substructure and relatedness were adjusted for by including the country, the top three country-specific principal components (PCs), and the genetic relationship matrix (GRM).

Although isolated BL cases are observed in rare Mendelian disorders [16–18], the genetic predisposition to sporadic BL has not been well studied. To investigate germline risk factors for BL, we enrolled 575 BL cases and 3,645 BL-free community controls from high-burden regions of Uganda, Tanzania, and Kenya [19], and 225 BL cases and 210 non-BL cancers in Malawi [20] (Figs. 1a, b and S1). Using this resource, we have characterized population structure in the SSA BL belt [21]; reported a BL-protective association for the *HBB-*rs334-T allele [22, 23] and BL-risk associations for the *HLA-DQA1-*rs2040406-G allele [24] and mosaic chromosomal alterations [25].

Here, using a genome-wide association study (GWAS), we report novel and robust BL-protective associations within chr21q22.12 tagged by the rs111457485-T allele located upstream of *RUNX1*. Genomic and functional studies suggest that the linked rs2242780-C allele is the putative functional variant accounting for this association.

## Methods

### Ethical approval and consent to participate in GWAS

We confirm that all relevant ethical guidelines and regulations were followed. The approval for the EMBLEM study was granted by ethics committees at the Uganda Virus Research Institute (UVRI, GC/127), the Uganda National Council for Science and Technology (HS 816), the Tanzania National Institute for Medical Research (NIMR/HQ/R.8c/Vol. IX/1023), Moi University/Moi Teaching and Referral Hospital (000536), and the US National Cancer Institute (10-C-N133). Permission to retrospectively recontact patients to determine the vital status of all 249 Ugandan BL patients included in the GWAS was obtained from UVRI (GC/127/178). The original ethical approval for the Infections and Childhood Cancer Study in Malawi was granted by ethics committees at the Malawi College of Medicine (P.03/04/277 R) and Oxford University. Permission to conduct genetic testing on residual samples from Malawi was obtained from the Malawi National Health Sciences Research Committee in 2019 (Approval #2405). Written informed consent was obtained from the participants’ guardians in the EMBLEM and Malawi studies, and written informed consent was obtained from children aged ≥7 years in the EMBLEM study. Ethical approval for GWAS was granted on the stipulation that research focused on the established risk factors for BL, including malaria, malaria resistance genes [23], and *HLA* variation [24], was prioritized.

The study methods have been reported elsewhere [14, 19, 20] and are described in the Supplementary Information. Briefly, DNA extracted from buffy coats or saliva was staged and genotyped at the National Cancer Institute Cancer Genomics Research Laboratory [21, 23, 24]. The resulting 2,267,535 high-quality SNPs were phased and imputed with EAGLE2 [26] and PBWT [27], respectively, using the African Genome Resources (AGR) as a reference panel (imputation.sanger.ac.uk). Principal component analysis (PCA) [28] was performed based on 787,731 independent SNPs (*r*^*2*^ < 0.3) to assess population structure in the combined set (Fig. S2) and separately for each country to calculate population-specific principal components (PCs, Fig. S3) used to adjust for genomic variation. GWAS with loci was performed in the combined set using loci with INFO > 0.3 and minor allele count >20 by fitting logistic mixed model regression in SAIGE [29] (version 1.1.6.2), controlling for age, sex, country, the top three population-specific PCs as fixed-effects, and the genetic relationship matrix (GRM) as a random effect. Adjusting for genetic relationships allowed keeping related individuals in the dataset. Leave-one-chromosome-out (LOCO) model fitting was enabled to prevent proximal contamination. A *p* < 5 × 10^⁻8^ denotes genome-wide significance, while a *p* > 5 × 10^⁻8^ < *p* < 5 × 10^⁻7^ is suggestive. The association results are presented as odds ratios (ORs) and 95% confidence intervals (95% CIs).

Several sensitivity analyses were performed on the 21q22.12 association. The GWAS results for the combined dataset were compared to those from a standard meta-analysis of the country-specific ORs, and the heterogeneity in the country-specific results was evaluated using Cochran’s Q test. Additionally, the GWAS results for the combined dataset were compared to those obtained after removing individuals with population-specific PCs >1.5 * interquartile range as outliers (*n* = 346, Fig. S4) and individuals who were first- or second-degree relatives (*n* = 609), based on KING to generate a maximal independent set. Additional analyses were performed controlling for factors previously associated with BL risk (*P. falciparum* positivity [19], *HBB*-rs334-T [23] and *HLA-DQA1-*rs2040406-G alleles [24], plasma EBV DNA copy number and EBV antibody titers [30]). Stratified analyses for BL risk were performed by age (< or ≥9 years) to assess effects based on the age when naturally acquired immunity to malaria peaks [31] and tumor anatomic site (head-only, *n* = 177 or abdominal-only, *n* = 253) compared with all the controls.

Fine-mapping of GWAS loci was performed using the Sum of Single Effects (SuSiE) model to identify a set of SNPs with a 95% probability of including the causal variant(s), hereafter referred to as the credible set SNPs (CS-SNPs). The ancestral patterns of the CS-SNPs were evaluated in the long-read whole-genome sequencing (WGS) assemblies of the Human Pangenome Reference Consortium (HPRC) [32] and in archaic humans as described elsewhere [33]. The associations between the CS-SNPs and total and isoform-level RNA expression in African BL tumors were evaluated in the BL Genome Sequencing Project (BLGSP) dataset [14] using linear regression models adjusting for age, sex, and tumor EBV status (positive/negative).

Cis-acting expression quantitative trait loci (eQTLs) were queried in transcriptomic databases, including eQTLGen (whole blood, *n* = 31,355), the Genotype-Tissue Expression (GTEx) dataset (54 tissues from 838 donors), MuTHER lymphoblastoid cell lines (LCLs, *n* = 856) [34], and PBMCs (*n* = 1012) of controls of African ancestry in the Jackson Heart Study (JHS) [35]. Genotyping of 55 BL-derived cell lines identified one (EB-3, cultured from BL in a Ugandan child [36]) that carried the effect alleles of chr21q22.12 SNPs. Pore-C analysis was performed for EB-3, Raji, and DG-75 BL-derived cell lines to investigate long-range interactions with the regions harboring the BL GWAS SNPs. The potential regulatory activity of the GWAS loci was investigated using Luciferase reporter assays in the EB-3, Raji, HEK293T (an embryonal kidney cell line) cells. The fold differences in the allele-specific Luciferase results were compared using two-sided unpaired *t*-tests. In silico prediction of transcription factor-binding sites was done using online tools. DNA-protein interactions were evaluated by electrophoretic mobility shift assays (EMSAs).

Associations between the CS-SNPs and somatic mutations in African BL tumors were evaluated in the BLGSP [13] by linear regression models. Tumor mutational burden (TMB) [13] was defined as the sum of tumor-specific single base substitutions and small (<50 base pairs) insertions/deletions in coding and non-coding regions [13]. Four COSMIC mutational signatures previously identified in the BLGSP BL tumors [13] were analyzed: signature A (SBS5, age-associated), signature B (SBS17 with unknown etiology), signature C (SBS15 associated with defective DNA mismatch repair), and signature D (SBS9 associated with AICDA and polymerase η activity).

Overall survival (OS) was analyzed in BL patients from Uganda with available vital status using the log-rank test and Kaplan‒Meier and Cox proportional hazard regression models. These analyses controlled for age, sex, plasma EBV positivity (yes/no), number of chemotherapy drugs administered (0 for untreated and 1–6 drugs), and tumor anatomic site (head-only, abdominal-only, head and abdominal and other).

Multi-marker association analysis was performed using the MAGMA package (version 1.08) [37] and transcriptome-wide association study (TWAS) analysis using FUSION (https://gusevlab.org/projects/fusion/) with the trained GTEx v8 models.

## Results

### Epidemiological characteristics of BL

The participant characteristics are summarized in Figs. 1a, b and S5a–j. BL patients were predominantly males (Fig. S5b), as expected [38, 39]. The mean age was ~7 years in both cases and controls (Fig. S5c), indicating comparable cumulative exposure to malaria [8]. However, current or recent *P. falciparum* infection was less common in cases than in controls (18.9%/5.4% versus 33.2%/15.3%, *p* < 0.0001, Fig. S5g), likely due to greater suppression of parasitemia in cases leading to submicroscopic infection and false-negative results [40]. By anatomic site, *P. falciparum* infection prevalence was greater in patients with head-only than in those with abdominal-only BL (48% versus 39%, *p* = 0.001; Fig. S5h). BL was predominantly abdominal—alone (49.4%, *n* = 253) or with head involvement (12.5%, *n* = 64), followed by head-only (34.6%, *n* = 177), and disseminated sites (2.5%, *n* = 18). As expected [38, 39], head-only BL predominated in males (38% versus 27% in females, *p* = 0.004; Fig. S5i). The mean age at diagnosis varied slightly for BL involving different anatomic sites (Fig. S5j).

### Population structure

Population structure was observed among study participants overall, but not among the cases and controls (Fig. S2). Significant genetic relatedness was observed among the controls, particularly in Uganda (Fig. S3), but only two BL cases were related [41]. ADMIXTURE analysis of genetic ancestry revealed predominantly Nilotic ancestry (60–90%) in participants in northern Uganda [21], predominantly Bantu ancestry (80–90%) in those from Malawi and northern Tanzania, and 30:70% Nilotic:Bantu ancestries in those from Kenya and Tanzania (Fig. 1c).

### GWAS results

**Fig. 2:**
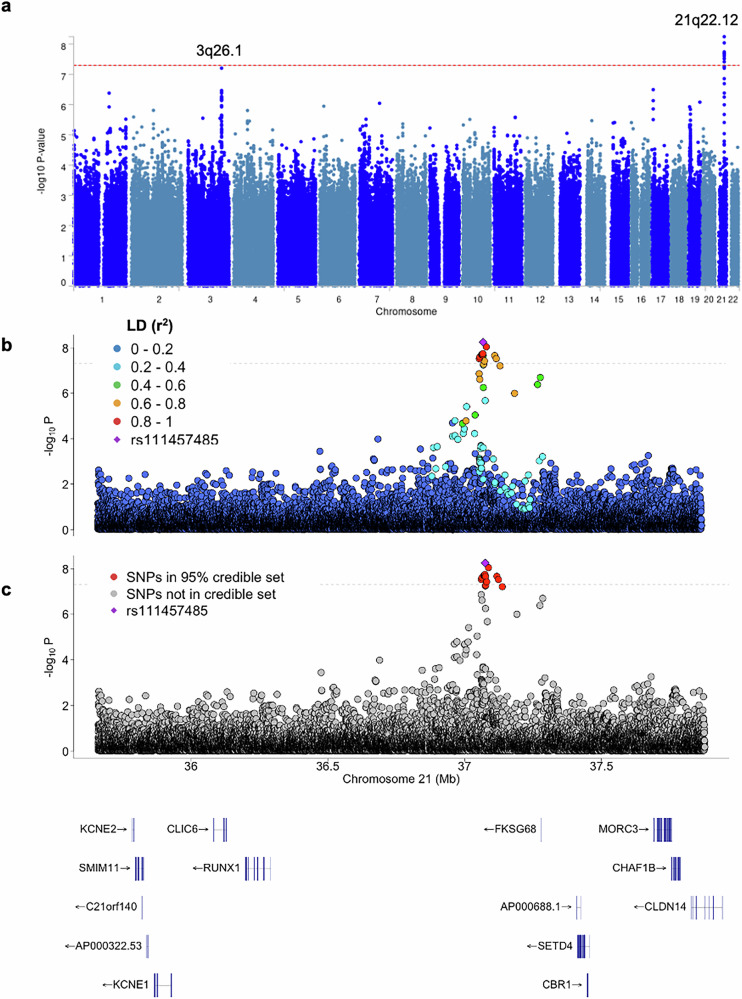
BL GWAS association results. **a** Manhattan plot presenting the results of the BL GWAS of 800 patients and 3845 controls. The dotted line represents the genome-wide significance threshold (−log10 *p* = 5 × 10^−8^). **b** Regional plot of the chr21q22.12 BL GWAS locus located within a ~ 1 Mb gene desert between the *RUNX1* and *SETD4* genes; the index SNP rs111457485 (OR = 0.57; *p* = 5.7 × 10^−^^9^) is marked in magenta. The markers are colored based on LD (*r*^2^) with rs111457485 in the total GWAS set (*N* = 4625). **c** 95% credible set for the chr21q22.12 BL GWAS locus comprising 17 credible set SNPs from fine-mapping analysis using SuSiE.

A novel BL-protective association was identified within chr21q22.12 (Fig. 2a) at a low genomic inflation factor (*λ*_GC_ = 1.006; Fig. S6). This association was tagged by the rs111457485-T allele with a MAF of 7.2% in cases and 12.2% in controls (OR = 0.57, 95% CI: 0.47–0.68, *p* = 5.7 × 10^−9^) and 12 other correlated SNPs (Fig. 2a, b; Table S1). No other variant within the chr21q22.12 region was significantly associated with BL after conditioning on rs111457485 (Table S2). The GWAS from a standard meta-analysis yielded similar results (OR = 0.57, 95% CI 0.47–0.69, *p* < 1.6 × 10^−8^) with no evidence of heterogeneity in country-specific ORs (*I*^2^ = 0.0, *p*_*het*_ = 0.57, Fig. S7). The results were similar in GWAS performed after removing PC outliers (OR = 0.58, 95% CI 0.47–0.71, *p* < 7.36 × 10^−8^) or outliers and related individuals (OR = 0.58, 95% CI 0.48–0.72, *p* < 1.81 × 10^−7^, Table S2).

The BL association with rs111457485-T was not confounded by *HBB*-rs334-T (OR = 0.56, *p* = 4.68 × 10^−^^9^), *HLA-DQA1-*rs2040406-G (OR = 0.58, *p* = 3.19 × 10^−^^8^), *P. falciparum* infection [19, 42] (OR = 0.57, *p* = 1.06 × 10^−^^8^), age (<9 years, OR = 0.58, *p* = 7.37 × 10^−6^ versus ≥9 years OR = 0.54, *p* = 1.26 × 10^−4^, *p*_het_ = 0.11), plasma EBV copies (OR = 0.60, *p* = 0.056, *n* = 737) or EBV antibodies OR = 54, *p* = 3.36 × 10^−4^, *n* = 1177, Table S3).

### Fine mapping

**Fig. 3:**
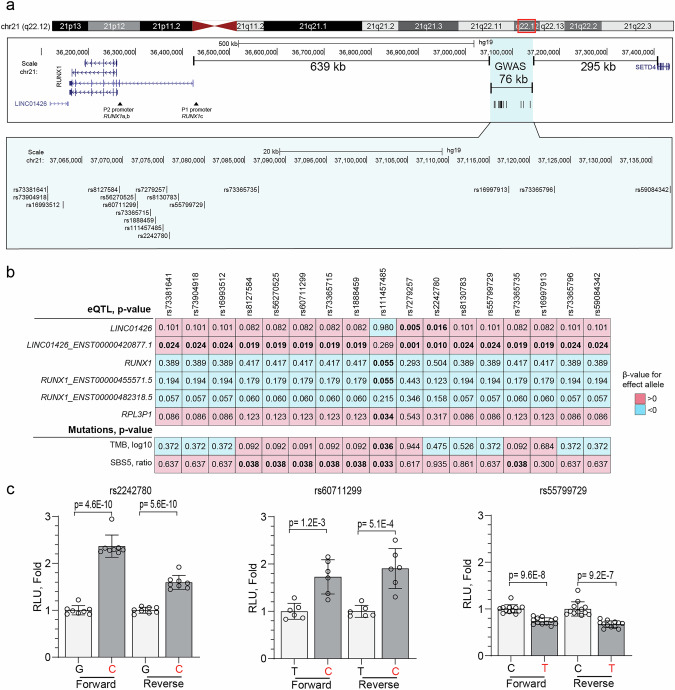
Genomic profile of the chr 21q22.12 BL GWAS locus. **a** Overview of the ~1 Mb region within the chr 21q22.12 locus, with a zoomed-in view of the 76 kb region comprising 17 CS-SNPs associated with BL risk; **b** Heatmap showing *p* values for the relationships between the 17 CS-SNPs and gene and isoform-level expression in the region in BL tumors (*n* = 86) and the total mutational burden (TMB) and the COSMIC SBS5 mutational signature in BL tumors (*n* = 89) of children from Uganda. Significant *p* values are bolded. The pink and blue colors represent the directions of *β* values for the effect alleles of all the SNPs. *p*-values and β values were derived from linear regression models adjusted for sex, age, and EBV status. Plots for select associations are shown in Fig. S10. **c** Results of Luciferase reporter assays in HEK293T cells identified significant regulatory activity for three of the 17 CS-SNPs tested, with allele-specific differences in both orientations. The results for the same SNPs in a BL-derived cell line (Raji) are shown in Fig. S12. The effect alleles of each variant are in red. Each transfection was performed in 6–12 technical replicates and repeated in 2–3 independent experiments with similar results. The results from one representative experiment are shown, with individual values, group means, and error bars representing standard deviation. *p*-values were calculated via an unpaired two-sided *t*-test.

Fine mapping of the chr21q22.12 locus identified a 76.6 kb genomic region with 17 BL-associated CS-SNPs with ORs ranging 0.55–0.65 (Fig. 2c, Table S4). This region is an ~1 Mb gene desert flanked by *RUNX1* and *SETD4* genes (Figs. 3a and S8). Based on the 1000 Genomes reference panel, the effect (minor) alleles of the 17 chr21q22.12 CS-SNPs are relatively common (10.7–15%) in African ancestry populations but rare (<1%) or absent in other ancestries (Table S5). These variants were identified only in three individuals (all African-ancestry) in the HPRC long-read assemblies [32], all carrying minor alleles of all chr21q22.12 CS-SNPs (Table S5). The effect alleles of two CS-SNPs (rs111457485-T and rs73365715-T) are ancestral variants found in chimpanzee and archaic humans, whereas 15 of the remaining CS-SNPs are human-specific (Table S5). Analysis of the long-read WGS in the EB-3 cell line (with the effect alleles of the chr21q22.12 CS-SNPs) and HPRC [32, 33] and short-read WGS in the BLGSP [14] did not detect other relevant SNPs or structural variants within the ~1.5 Mb region (Figs. 3a and S8) that could be linked with or explain the observed BL association.

### Expression quantitative trait loci (eQTL) analysis

No eQTLs were detected for the chr21q22.12 CS-SNPs in the GTEx dataset, perhaps due to the limited number of African ancestry donors (*n* = 122, 12.7%) [34]. However, the analysis of transcriptomes of PBMCs from African American persons in the JHS (*n* = 1012) [35] revealed significant associations of effect alleles of most CS-SNPs with increased expression of *RUNX1-AS* (ENSG00000286153), a long noncoding *RUNX1* antisense transcript, and with *DOPEY2* (ENSG00000142197), which encodes a DOP1 leucine zipper-like protein B (DOP1B) (Table S6). These transcripts are located, respectively, ~850 kb upstream and ~470 kb downstream of the chr21q22.12 CS-SNPs. These findings in the PBMC transcriptomes from controls were not replicated in the BLGSP data [13, 14], in which *RUNX1-AS* expression was not detected and *DOPEY2* expression was not associated with any of the CS-SNPs. However, the rs111457485-T allele was suggestively associated with decreased total *RUNX1* expression (*p* = 0.055, beta = −3.23 TPM, Fig. 3b) and decreased *RUNX1*-ENST00000455571.5 isoform expression (*p* = 0.055, beta = −1.02 TPM; Fig. S9), both regulated by the distal *RUNX1*-P1 promoter. The effect alleles of several other CS-SNPs (*p* = 0.055, beta = −1.02 TPM) were also associated with decreased expression of a *RUNX1*-ENST00000482318.5 isoform, which is also regulated by the *RUNX1*-P1 promoter.

In the BLGSP, the effect alleles of most CS-SNPs (but not rs111457485-T) were associated with significantly increased expression of total *LINC01426* or its isoform *LINC01426*-ENST00000420877.1 (Fig. 3b). This finding could not be evaluated in the PBMC transcriptomes in the JHS because *LINC01426* expression was not measured. *LINC01426* is a long noncoding RNA that is located immediately downstream of the *RUNX1* 3′UTR and is reported to affect *RUNX1* expression [43]. There was a significant correlation (*r* = 0.28, *p* = 0.006) between the expression of *LINC01426* and the *RUNX1-*ENST00000344691.8 isoform regulated by the P2 promoter, which also regulates *RUNX1a* and *RUNX1b* isoforms. However, no correlation was observed with *RUNX1c* isoforms from the P1 promoter. Most *RUNX1*-P1 transcripts from the OCI-Ly7 B-cell lymphoma cell line were full-length and thus can encode the RUNX1c protein (Fig. S10). In CRISPR perturbation and RNAi cell viability screens (DepMap portal), *RUNX1* was identified as a strongly selective/high-dependency gene, especially in B-lymphoblastic leukemia/lymphoma cell lines. Thus, any factors decreasing *RUNX1* expression, perhaps including the BL-protective alleles of the chr21q22.12 CS-SNPs, may decrease viability of the affected cells, which is an important functional outcome, but decrease the ability to detect allelic expression differences.

There was consistent overlap of DNA methylation dips (a correlate with RNA expression [44]) across the *RUNX1-*P1 and P2 promoter regions in B-cell tumors (Fig. S8) and with super-enhancers associated with several B-cell lymphomas [45]. The overlapping regions exhibited differential methylation and gene expression patterns between BL, follicular lymphoma, and normal GC B-cell populations (Fig. S8) [44], but these regions did not co-localize with the chr21q22.12 CS-SNPs.

### CS-SNPs as putative RUNX1 enhancers

The expression of Luciferase reporters was increased ~2-fold by the rs2242780-C and rs60711299-C alleles and decreased ~25% by the rs55799729-T allele (Fig. 3c), but it was not affected by other chr21q22.12 CS-SNPs. Similar results were obtained in Raji cells, but did not reach statistical significance, likely because of greater technical variation (Fig. S11a,b) due to the lower transfection efficiency (25%) than in HEK293T cells (49%). The EMSA results in nuclear cell extracts from HEK293T, Raji, and EB-3 cells revealed allele-specific binding for the rs2242780-C but not for the rs60711299-C or rs55799729-T alleles (Fig. S12). This is consistent with enhancer regulatory activity [46] for some of the chr21q22.12 CS-SNPs.

In silico analysis predicted stronger interaction for the rs2242780-C allele with STAT1/STAT3 proteins that regulate a range of biological functions related to immunity and tumor suppressor activity through the JAK/STAT pathway (Table S7) [47]. The rs55799729-T allele was predicted to have stronger interaction with GFI1B, which functions as a transcriptional repressor and promotes growth arrest and apoptosis in lymphomas [48]. The rs60711299-C allele was predicted to have stronger interaction with *ARID3A*, which modulates gene expression in B-cells [49].

Pore-C analysis revealed putative long-range (>700 kb) interactions in EB-3 cells. Specifically, a 2 kb region spanning the rs111457485 and rs2242780 loci showed interactions with the regions close to the *RUNX1-P1* or *LINC01426* promoters. However, these interactions were not detected in the DG-75 and Raji BL-derived cell lines (Fig. S13), which may reflect biological variation in cells lacking the effect alleles of the chr 21q22.12 CS-SNPs. Analysis of public ChIP-seq datasets revealed binding sites for the EBV proteins EBNA1, EBNA2, and EBNA3C in several BL-derived cell lines within the *RUNX1*-P1 promoter region (Fig. S13), but none of these colocalized with the chr21q22.12 BL GWAS locus.

### Associations of GWAS loci with clinical features

**Fig. 4:**
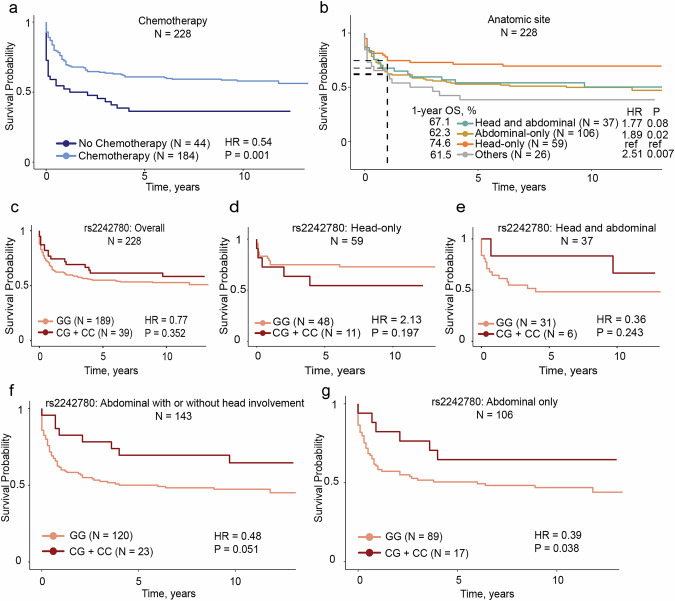
Analysis of overall survival (OS) in 228 BL patients from Uganda. The probability for overall survival (OS) is presented as unadjusted hazard ratios (HRs) and *p*-values (*p*) for OS by: **a** chemotherapy (reference: no chemotherapy) and **b** anatomic tumor sites (reference: head-only BL). HRs are for the entire period, with the 1-year OS probabilities reported in the text are shown by dashed lines on the *Y*-axis. *p*-values were calculated from the unadjusted Cox models using (**a**) chemotherapy and (**b**) anatomic sites as categorical variables. Multivariable Cox regression analysis of OS controlling for age, sex, EBV status and the number of chemotherapy drugs (0–6) in relation to rs2242780 (reference: GG genotype group, C allele is protective from BL in GWAS); **c** in the overall set (*n* = 228) with vital status; **d** In a subset of 59 BL patients with head-only tumors; **e** In a subset of 37 BL patients with head and abdominal tumors; **f** In a subset of 143 BL patients with abdominal tumors with or without head involvement (combining abdominal-only and head and abdominal tumors; **g** In a subset of 106 BL patients with abdominal-only tumors (Table S12).

Carriage of the rs111457485-T allele, but not the other CS-SNPs, was associated with increased tumor mutational burden (TMB, *p* = 0.036, Fig. 3b, Table S8). The rs111457485-T allele was also associated with an elevated ratio of COSMIC signature 5 (SBS5, *n* = 89, *p* = 0.033; Fig. 3b, Table S8). Compared with all the controls (*n* = 3845), BL-protective associations were observed for all the chr21q22.12 CS-SNPs with BL involving head-only (*n* = 177) or abdominal-only sites (*n* = 253) (Table S9). Among 228 BL patients with available vital status (including 106 deaths, Fig. S14a), 81.9% (*n* = 159) received one cycle out of three to six recommended first-line combination chemotherapy consisting of cyclophosphamide, vincristine, and methotrexate (COM). Chemotherapy was not recorded for 44 patients. Receiving chemotherapy significantly reduced deaths (57.6% alive vs. 36.3% dead, *p* = 0.017; 184 participants, 78 deaths; Table S10), resulting in a lower hazard ratio (HR) of death (HR = 0.54, *p* = 0.001; Fig. 4a). The 1-, 3-, and 5-year overall survival (OS) probabilities were 66.2%, 59.6% and 56.1%, respectively (Fig. S14b; Table S11). Among the patients who died, the median OS was 0.4 years (~4.8 months). OS did not vary by calendar year of enrollment (Fig. S15), plasma EBV positivity or sex [1] (Fig. S15a, b). The 1-year OS was 74.6% in patients with head-only tumors. Compared to this group, OS was intermediate (62–64%) in those with abdominal-only tumors (HR = 1.89, *p* = 0.020) and those with any abdominal tumors (HR = 1.77, *p* = 0.08) and lowest (42.9%) in those with disseminated BL (HR = 2.51, *p* = 0.007, Fig. 4b).

Although the BL-protective GWAS associations with the chr21q22.12 CS-SNPs effect alleles might reflect the survival bias of BL patients, this hypothesis was not supported by our data (Table S11). The strongest association with better OS was observed for the rs2242780-C allele (Fig. 4c), which also had the strongest functional data. In carriers of the rs2242780-C allele, OS was worse, albeit not significant, for patients with head-only BL (HR = 2.13, *p* = 0.197, 59 patients, 18 deaths; Fig. 4d). However, it was better for patients with abdominal-only BL (HR = 0.39, *p* = 0.038; 106 patients, 59 deaths) and in those with any abdominal BL involvement (HR = 0.48, *p* = 0.051; 143 patients, 77 deaths; Fig. 4e–g). Unexpectedly, the OS appeared to be worse in carriers of the rs111457485-T allele with head-only tumors (HR = 3.84, *p* = 0.072; 59 participants, 18 deaths; Fig. S15d).

### Suggestive GWAS loci and gene-aggregated results

We observed a BL-protective association within the chr3q26.1 locus tagged by the rs9847876-C allele (OR = 0.71, 95% CI = 0.62–0.80, *p* = 6.2 × 10^−8^; Fig. 2a), with a MAF of 33.1% in cases and 42.3% in controls (Table S1; Fig. S16a). Fine-mapping of the chr3q26.1 locus identified 48 CS-SNPs within a 21.7 kb genomic region (Table S4, Fig. S16b) located near the *OTOL1* gene, which encodes a member of the C1q/tumor necrosis factor-related protein (CTRP) family. Analysis of eQTLs showed consistent associations of the rs9847876-C allele with increased expression of *NMD3* (encoding a ribosome export adapter). The eQTLs with *NMD3* were detected both in the whole blood of European donors in eQTLGen (n = 31,355, *p* = 6.7 × 10^−21^) and in the PBMCs of African American donors in the JHS (*n* = 1012, *p* = 1.9 × 10^−3^) (Table S12).

MAGMA analysis identified signals within the chr3q26.1 (*OTOL1*; *p* = 1.7 × 10^−7^) and chr19p13.2 (*C19orf82*; *p* = 2.3 × 10^−6^) loci (Fig. S17, Table S13). TWAS in whole blood and splenic tissue in GTEx.v8 (the tissues most relevant for BL) identified associations with genetically regulated expression within the chr19p13.2 for *FBXL12* (*p* = 2.3×10^-6^) in whole blood and *UBE2L4* (*p* = 5.4×10^-06^) and *lnc-UBL5-L5* (*p* = 1.1 × 10^-5^) in the spleen (Table S13).

## Discussion

By performing a GWAS in cases and controls from four East African countries with high malaria/BL burden, we discovered novel significant BL-protective association within the chr21q22.12 locus marked by the rs111457485-T allele (OR = 0.57; *p* = 5.7 × 10^−9^). The GWAS results were robust in standard meta-analysis and in sensitivity analyses (excluding PC outliers and related individuals) and in analyses adjusting for factors associated with BL (*P. falciparum* or EBV infections, age, *HBB*-rs334-T, and *HLA-DQA1-*rs2040406-G). Fine mapping identified 17 CS-SNPs, including rs2242780 for which our analysis identified relevant functional properties. These loci near *RUNX1*, a gene implicated in myeloid malignancy [50, 51], provide novel clues about the genetics of BL.

To glean some insights about the GWAS findings, we used limited genomic and transcriptomic datasets enriched for African subjects (the BLGSP [13, 14], PBMC transcriptomes of controls in the JHS [35]), as well as methylomes of BL, other lymphomas, and normal GC B-cell populations [44], followed by in vitro functional studies in cell lines. From these analyses we infer that *RUNX1* is the most likely gene underlying the BL GWAS association in the chr21q.22.12 region. *RUNX1* expression is critical to the physiology of GC B-cells [10], from which BL originates. High *RUNX1* expression is associated with a resting state of GC B-cells [52], while low expression is associated with a proliferative state, such as in EBV immortalized B-cells [52] or BL tumors [53]. The BL GWAS chr21q.22.12 locus is located near the genomic regions with B-cell lymphoma-associated superenhancers [45], which are differentially methylated between BL, follicular lymphoma, and normal GC B-cell populations [44]. The long-range chromatin interactions detected between the locus harboring the BL CS-SNPs and the *RUNX1* region ~700 kb away support the hypothesis that one or several of the CS-SNPs help to form and maintain long-range interaction loops facilitating the regulation of *RUNX1*. Specifically, a 2-kb fragment harboring both rs111457485 and rs2242780 showed chromatin interactions with a region close to the distal *RUNX1-*P1 promoter. The strong Luciferase reporter results in HEK293T cells support the inference of enhancer activity of the rs2242780-C allele. The results were weaker but consistent in Raji and DG-75 cells. The EMSA results were consistent with the preferential binding of nuclear proteins to the rs2242780-C allele in the three cell lines. It is plausible that rs2242780, which is linked to rs111457485 (*r*^*2*^ = 0.69 in GWAS controls), is the functional variant of the chr21q22.12 locus. The effects attributed to *RUNX1* might be also contributed by *DOPEY* or *LINC01426*. The *RUNX1-*P1 promoter regulates the expression of *RUNX1* and the *RUNX1c* isoform, and EBV is known to downregulate *RUNX1* expression [53] by inhibiting the *RUNX1*-P1 promoter via EBNA2, EBNA3B and EBNA3C encoded transcription factor binding [53]. The regulation of *RUNX1*c transcription may be a common pathway controlling B-cell proliferation that links BL risk with EBV or host germline factors, such as in the chr21q22.12.

Our results may be relevant for BL outside Africa. In the US, about 10% of BL cases occur in African Americans [3, 54], but with lower relative risk for sporadic BL (decreased by ~25% lower) [54, 55], HIV-[56] and post-transplant [57] associated BL (~60–70% lower), despite a greater burden of EBV infection from an early age compared to European Americans [58]. Given the recent consensus that EBV defines molecular subtypes of BL in all geographical settings [59], the lower risk of BL in African Americans is intriguing. The BL-protective chr21q.22.12 variants, which are strongly enriched in populations of African ancestry (Fig. S18), might contribute to this BL pattern. Furthermore, in 30,762 African American participants in the Million Veteran Program [60], we noted association of elevated serum ferritin levels with BL-protective alleles—rs111457485-T (*β* = 0.042, *p* = 3 × 10^−3^) and rs2242780-C (*β* = 0.05, *p* = 1.1 × 10^−4^), suggesting that the chr21q22.12 loci may affect iron homeostasis. A mendelian randomization study among European individuals suggested that elevated serum ferritin is protective from lymphoma risk [61]. The link between the BL-protective chr21q.22.12 variants and lymphoid malignancies, for which risks are lower in African- compared to European-ancestry individuals [62–64], warrants further investigation.

Our findings of different OS by anatomic site are reminiscent of the distinct epidemiological patterns of BL by anatomic site [38, 39]. Notably, a younger age at onset (~3–5 years) and male predominance in BL cases involving the head-only [65] versus an older age at onset (~7–9 years) and female predominance in abdominal BL cases. The greater *P. falciparum* positivity in head-only compared with abdominal-only BL cases in our data is another epidemiological clue about biological heterogeneity of BL by anatomic site.

The strengths of our study include using well-characterized samples from four African countries with high malaria and BL burden [3], and integrating trans-disciplinary methods to probe unique genetic, genomic, and transcriptomic datasets for follow-up studies. Our finding of lower *P. falciparum* infection in BL cases compared to controls is counterintuitive given the strong correlation between BL risk and cumulative malaria infections [8], but in line with our studies [19, 23] and reports by others [66, 67]. This pattern may be explained by postulating stronger naturally acquired immunity (NAI) in semi-immune individuals, also referred to as premunition [31], in BL cases than controls [23]. NAI is necessary for suppression of *P. falciparum* parasitemia [68], which may result in false *P. falciparum* negativity in semi-immune people with low-density or submicroscopic infections [40]. BL cases are 2-fold more likely to have submicroscopic (*P. falciparum* PCR positive) infections and 2.4-fold more likely to have mixed genotype infections [69, 70]. These findings suggest that submicroscopic/mixed genotype infections in semi-immune children [71] are relevant risk factors of BL, likely by driving chronic lytic EBV infection [72] (Fig. S19).

The limitations of our study include the relatively small GWAS sample size and lack of a replication cohort. By using Bonferroni correction and multiple analytic methods, the likelihood of false-negative results was reduced, while less stringent criteria and multi-marker and gene-aggregation enabled us to identify promising leads, such as within 3q26.1 and 19p13.2 regions (Table S13), which may be investigated in future studies. The inclusion of participants only from East Africa may affect the generalizability of the results, but our successful GWAS should encourage research on BL genetics to replicate and extend our findings. Our OS results showing low survival rate of BL echo those from elsewhere in Tanzania [73], Kenya [74, 75] and Cote d’Ivoire [76]. Such low survival rate of BL in African patients contrasts sharply with the >90% survival rates reported in Europe and North America [1]. The reasons for the poor outcomes in Africa include delayed or imprecise diagnosis due to challenges in timely access to high-quality hematopathology [77] and unreliable access to chemotherapy.

In conclusion, we report a novel BL-protective locus on chr21q22.12 near *RUNX1* for BL in African children. Our work advances the understanding of the genetic basis of BL, highlights the feasibility and scalability of BL GWAS in Africa, and identifies functional loci with potential clinical impact on BL.

## Supplementary information

Supplementary Material

Tables S1-S15

## Data Availability

The genetic data reported in this paper are available through dbGaP under accession code phs001705.v1.p1 (EMBLEM), and the BLGSP files can be accessed from the Genomic Data Commons (GDC, https://portal.gdc.cancer.gov/; Project ID: CGCI-BLGSP, dbGaP study accession: phs000527.v13.p4). DepMap data for RUNX1 were accessed at https://depmap.org/portal. The Pore-C and whole-genome sequencing data for BL cell lines generated in this study have been deposited in the NCBI with a BioProject accession number (PRJNA1212064). Covariate data for EMBLEM can be applied for directly by request to the corresponding author (SMM).

## References

[1] López C, Burkhardt B, Chan JKC, Leoncini L, Mbulaiteye SM, Ogwang MD, et al. Burkitt lymphoma. Nat Rev Dis Prim. 2022;8:78. 36522349 10.1038/s41572-022-00404-3

[2] Linet MS, Brown LM, Mbulaiteye SM, Check D, Ostroumova E, Landgren A, et al. International long-term trends and recent patterns in the incidence of leukemias and lymphomas among children and adolescents ages 0-19 years. Int J Cancer. 2016;138:1862–74. 26562742 10.1002/ijc.29924 PMC12439319

[3] Mbulaiteye SM, Devesa SS. Burkitt lymphoma incidence in five continents. Hemato. 2022;3:434–53.

[4] Epstein MA, Achong BG, Barr YM. Virus particles in cultured lymphoblasts from Burkitt’s lymphoma. Lancet. 1964;1:702–3. 14107961 10.1016/s0140-6736(64)91524-7

[5] Bornkamm GW. Epstein-Barr virus and the pathogenesis of Burkitt’s lymphoma: more questions than answers. Int J Cancer. 2009;124:1745–55. 19165855 10.1002/ijc.24223

[6] Aka P, Vila MC, Jariwala A, Nkrumah F, Emmanuel B, Yagi M, et al. Endemic Burkitt lymphoma is associated with strength and diversity of Plasmodium falciparum malaria stage-specific antigen antibody response. Blood. 2013;122:629–35. 23645841 10.1182/blood-2012-12-475665 PMC3731925

[7] Dalldorf G, Linsell CA, Barnhart FE, Martyn R. An epidemiologic approach to the lymphomas of African children and Burkitt’s sacroma of the jaws. Perspect Biol Med. 1964;7:435–49. 14201808 10.1353/pbm.1964.0023

[8] Broen K, Dickens J, Trangucci R, Ogwang MD, Tenge CN, Masalu N, et al. Burkitt lymphoma risk shows geographic and temporal associations with Plasmodium falciparum infections in Uganda, Tanzania, and Kenya. Proc Natl Acad Sci USA. 2023;120:e2211055120. 36595676 10.1073/pnas.2211055120 PMC9926229

[9] Torgbor C, Awuah P, Deitsch K, Kalantari P, Duca KA, Thorley-Lawson DA. A multifactorial role for P. falciparum malaria in endemic Burkitt’s lymphoma pathogenesis. PLoS Pathog. 2014;10:e1004170. 24874410 10.1371/journal.ppat.1004170 PMC4038605

[10] Basso K, Dalla-Favera R. Germinal centres and B cell lymphomagenesis. Nat Rev Immunol. 2015;15:172–84. 25712152 10.1038/nri3814

[11] Ambrosio MR, Rocca BJ, Leoncini L. Burkitt Lymphoma. In: van Krieken JHJM, (ed). Encyclopedia of Pathology. Cham: Springer International Publishing; 2019. p. 1–12.

[12] Glaser S, Wagener R, Kretzmer H, Lopez C, Baptista MJ, Bens S, et al. Subtyping Burkitt Lymphoma by DNA Methylation. Genes Chromosomes Cancer. 2025;64:e70042. 40192513 10.1002/gcc.70042 PMC11974478

[13] Thomas N, Dreval K, Gerhard DS, Hilton LK, Abramson JS, Ambinder RF, et al. Genetic subgroups inform on pathobiology in adult and pediatric Burkitt lymphoma. Blood. 2023;141:904–16. 36201743 10.1182/blood.2022016534 PMC10023728

[14] Grande BM, Gerhard DS, Jiang AX, Griner NB, Abramson JS, Alexander TB, et al. Genome-wide discovery of somatic coding and noncoding mutations in pediatric endemic and sporadic Burkitt lymphoma. Blood. 2019;133:1313–24. 30617194 10.1182/blood-2018-09-871418 PMC6428665

[15] López C, Kleinheinz K, Aukema SM, Rohde M, Bernhart SH, Hübschmann D, et al. Genomic and transcriptomic changes complement each other in the pathogenesis of sporadic Burkitt lymphoma. Nat Commun. 2019;10:1459. 30926794 10.1038/s41467-019-08578-3 PMC6440956

[16] Chaigne-Delalande B, Li FY, O’Connor GM, Lukacs MJ, Jiang P, Zheng L, et al. Mg2+ regulates cytotoxic functions of NK and CD8 T cells in chronic EBV infection through NKG2D. Science. 2013;341:186–91. 23846901 10.1126/science.1240094 PMC3894782

[17] Purtilo DT, Szymanski I, Bhawan J, Yang JP, Hutt LM, Boto W, et al. Epstein-Barr virus infections in the X-linked recessive lymphoproliferative syndrome. Lancet. 1978;1:798–801. 85816 10.1016/s0140-6736(78)92999-9

[18] Zhukova N, Naqvi A. Williams-Beuren Syndrome and Burkitt Leukemia. J Pediatr Hematol Oncol. 2013;35:e30–32. 23018576 10.1097/MPH.0b013e318270672f

[19] Peprah S, Ogwang MD, Kerchan P, Reynolds SJ, Tenge CN, Were PA, et al. Risk factors for Burkitt lymphoma in East African children and minors: a case-control study in malaria-endemic regions in Uganda, Tanzania and Kenya. Int J Cancer. 2020;146:953–69. 31054214 10.1002/ijc.32390 PMC6829037

[20] Mutalima N, Molyneux E, Jaffe H, Kamiza S, Borgstein E, Mkandawire N, et al. Associations between Burkitt lymphoma among children in Malawi and infection with HIV, EBV and malaria: results from a case-control study. PLoS ONE. 2008;3:e2505. 18560562 10.1371/journal.pone.0002505 PMC2423475

[21] Gouveia MH, Bergen AW, Borda V, Nunes K, Leal TP, Ogwang MD, et al. Genetic signatures of gene flow and malaria-driven natural selection in sub-Saharan populations of the “endemic Burkitt Lymphoma belt”. PLoS Genet. 2019;15:e1008027. 30849090 10.1371/journal.pgen.1008027 PMC6426263

[22] Legason ID, Pfeiffer RM, Udquim KI, Bergen AW, Gouveia MH, Kirimunda S, et al. Evaluating the causal link between malaria infection and endemic Burkitt lymphoma in Northern Uganda: a mendelian randomization study. EBioMedicine. 2017;25:58–65. 29033373 10.1016/j.ebiom.2017.09.037 PMC5704046

[23] Hong HG, Gouveia MH, Ogwang MD, Kerchan P, Reynolds SJ, Tenge CN, et al. Sickle cell allele HBB-rs334(T) is associated with decreased risk of childhood Burkitt lymphoma in East Africa. Am J Hematol. 2024;99:113–23. 38009642 10.1002/ajh.27149 PMC10872868

[24] Liu Z, Luo Y, Kirimunda S, Verboom M, Onabajo OO, Gouveia MH, et al. Human leukocyte antigen-DQA1*04:01 and rs2040406 variants are associated with elevated risk of childhood Burkitt lymphoma. Commun Biol. 2024;7:41. 38182727 10.1038/s42003-023-05701-5 PMC10770398

[25] Zhou W, Fischer A, Ogwang MD, Luo W, Kerchan P, Reynolds SJ, et al. Mosaic chromosomal alterations in peripheral blood leukocytes of children in sub-Saharan Africa. Nat Commun. 2023;14:8081. 38057307 10.1038/s41467-023-43881-0 PMC10700489

[26] Loh PR, Danecek P, Palamara PF, Fuchsberger C, AR Y, KF H, et al. Reference-based phasing using the haplotype reference consortium panel. Nat Genet. 2016;48:1443–8. 27694958 10.1038/ng.3679 PMC5096458

[27] Durbin R. Efficient haplotype matching and storage using the positional Burrows-Wheeler transform (PBWT). Bioinformatics. 2014;30:1266–72. 24413527 10.1093/bioinformatics/btu014 PMC3998136

[28] Purcell S, Neale B, Todd-Brown K, Thomas L, Ferreira MA, Bender D, et al. PLINK: a tool set for whole-genome association and population-based linkage analyses. Am J Hum Genet. 2007;81:559–75. 17701901 10.1086/519795 PMC1950838

[29] Zhou W, Bi W, Zhao Z, Dey KK, Jagadeesh KA, Karczewski KJ, et al. SAIGE-GENE+ improves the efficiency and accuracy of set-based rare variant association tests. Nat Genet. 2022;54:1466–9. 36138231 10.1038/s41588-022-01178-w PMC9534766

[30] Coghill AE, Proietti C, Liu Z, Krause L, Bethony J, Prokunina-Olsson L, et al. The Association between the comprehensive Epstein-Barr virus serologic profile and endemic Burkitt lymphoma. Cancer Epidemiol Biomark Prev. 2020;29:57–62. 10.1158/1055-9965.EPI-19-0551 PMC6954331 31619404

[31] Smith T, Felger I, Tanner M, Beck HP. Premunition in Plasmodium falciparum infection: insights from the epidemiology of multiple infections. Trans R Soc Trop Med Hyg. 1999;93:59–64. 10450428 10.1016/s0035-9203(99)90329-2

[32] Liao W-W, Asri M, Ebler J, Doerr D, Haukness M, Hickey G, et al. A draft human pangenome reference. Nature. 2023;617:312–24. 37165242 10.1038/s41586-023-05896-x PMC10172123

[33] Florez-Vargas O, Ho M, Hogshead MH, Papenberg BW, Lee CH, Forsythe K, et al. Genetic regulation of TERT splicing affects cancer risk by altering cellular longevity and replicative potential. Nat Commun. 2025;16:1676. 39956830 10.1038/s41467-025-56947-y PMC11830802

[34] The GTEx Consortium atlas of genetic regulatory effects across human tissues. Science 2020 Sep 11; 369:1318-30. 10.1126/science.aaz1776 PMC7737656 32913098

[35] Wen J, Sun Q, Huang L, Zhou L, Doyle MF, Ekunwe L, et al. Gene expression and splicing QTL analysis of blood cells in African American participants from the Jackson Heart Study. Genetics. 2024;228:iyae098. 10.1093/genetics/iyae098 PMC11373511 39056362

[36] Epstein MA, Barr YM, Achong BG. Studies with Burkitt’s lymphoma. Wistar Inst Symp Monogr. 1965;4:69–82. 5884338

[37] de Leeuw CA, Mooij JM, Heskes T, Posthuma D. MAGMA: generalized gene-set analysis of GWAS data. PLoS Comput Biol. 2015;11:e1004219. 25885710 10.1371/journal.pcbi.1004219 PMC4401657

[38] Ogwang MD, Bhatia K, Biggar RJ, Mbulaiteye SM. Incidence and geographic distribution of endemic Burkitt lymphoma in northern Uganda revisited. Int J Cancer. 2008;123:2658–63. 18767045 10.1002/ijc.23800 PMC2574984

[39] Aka P, Kawira E, Masalu N, Emmanuel B, Brubaker G, Magatti J, et al. Incidence and trends in Burkitt lymphoma in northern Tanzania from 2000 to 2009. Pediatr Blood Cancer. 2012;59:1234–8. 22618958 10.1002/pbc.24194 PMC3427713

[40] Budodo R, Mandai SS, Bakari C, Seth MD, Francis F, Chacha GA, et al. Performance of rapid diagnostic tests, microscopy, and qPCR for detection of Plasmodium parasites among community members with or without symptoms of malaria in villages located in North-western Tanzania. Malar J. 2025;24:115. 40205516 10.1186/s12936-025-05361-2 PMC11984112

[41] Gouveia MH, Otim I, Ogwang MD, Wang M, Zhu B, Cole N, et al. Endemic Burkitt Lymphoma in second-degree relatives in Northern Uganda: in-depth genome-wide analysis suggests clues about genetic susceptibility. Leukemia. 2021;35:1209–13. 10.1038/s41375-020-01052-w PMC8024190 33051549

[42] Peprah S, Ogwang MD, Kerchan P, Reynolds SJ, Tenge CN, Were PA, et al. Inverse association of falciparum positivity with endemic Burkitt lymphoma is robust in analyses adjusting for pre-enrollment malaria in the EMBLEM case-control study. Infect Agent Cancer. 2021;16:40. 34099001 10.1186/s13027-021-00377-0 PMC8186042

[43] Wu H, Zheng J, Deng J, Zhang L, Li N, Li W, et al. LincRNA-uc002yug.2 involves in alternative splicing of RUNX1 and serves as a predictor for esophageal cancer and prognosis. Oncogene. 2015;34:4723–34. 25486427 10.1038/onc.2014.400

[44] Kretzmer H, Bernhart SH, Wang W, Haake A, Weniger MA, Bergmann AK, et al. DNA methylome analysis in Burkitt and follicular lymphomas identifies differentially methylated regions linked to somatic mutation and transcriptional control. Nat Genet. 2015;47:1316–25. 26437030 10.1038/ng.3413 PMC5444523

[45] Bal E, Kumar R, Hadigol M, Holmes AB, Hilton LK, Loh JW, et al. Super-enhancer hypermutation alters oncogene expression in B cell lymphoma. Nature. 2022;607:808–15. 35794478 10.1038/s41586-022-04906-8 PMC9583699

[46] Blobel GA, Higgs DR, Mitchell JA, Notani D, Young RA. Testing the super-enhancer concept. Nat Rev Genet. 2021;22:749–55. 34480110 10.1038/s41576-021-00398-w

[47] Wang W, Lopez McDonald MC, Kim C, Ma M, Pan ZT, Kaufmann C, et al. The complementary roles of STAT3 and STAT1 in cancer biology: insights into tumor pathogenesis and therapeutic strategies. Front Immunol. 2023;14:1265818. 38022653 10.3389/fimmu.2023.1265818 PMC10663227

[48] Xu W, Kee BL. Growth factor independent 1B (Gfi1b) is an E2A target gene that modulates Gata3 in T-cell lymphomas. Blood. 2007;109:4406–14. 17272506 10.1182/blood-2006-08-043331

[49] Hayakawa K, Li Y-S, Shinton SA, Bandi SR, Formica AM, Brill-Dashoff J, et al. Crucial role of increased Arid3a at the Pre-B and immature B cell stages for B1a cell generation. Front Immunol. 2019 ;15:457. 10.3389/fimmu.2019.00457 PMC6428705 30930899

[50] Cunningham L, Merguerian M, Calvo KR, Davis J, Deuitch NT, Dulau-Florea A, et al. Natural history study of patients with familial platelet disorder with associated myeloid malignancy. Blood. 2023;142:2146–58. 37738626 10.1182/blood.2023019746 PMC10733826

[51] Homan CC, King-Smith SL, Lawrence DM, Arts P, Feng J, Andrews J, et al. The RUNX1 database (RUNX1db): establishment of an expert curated RUNX1 registry and genomics database as a public resource for familial platelet disorder with myeloid malignancy. Haematologica. 2021;106:3004–7. 34233450 10.3324/haematol.2021.278762 PMC8561292

[52] Gunnell A, Webb HM, Wood CD, McClellan MJ, Wichaidit B, Kempkes B, et al. RUNX super-enhancer control through the Notch pathway by Epstein-Barr virus transcription factors regulates B cell growth. Nucleic Acids Res. 2016;44:4636–50. 26883634 10.1093/nar/gkw085 PMC4889917

[53] Brady G, Whiteman HJ, Spender LC, Farrell PJ. Downregulation of RUNX1 by RUNX3 requires the RUNX3 VWRPY sequence and is essential for Epstein-Barr virus-driven B-cell proliferation. J Virol. 2009;83:6909–16. 19403666 10.1128/JVI.00216-09 PMC2698531

[54] Mburu W, Devesa SS, Check D, Shiels MS, Mbulaiteye SM. Incidence of Burkitt lymphoma in the United States during 2000 to 2019. Int J Cancer. 2023;153:1182–91. 37278097 10.1002/ijc.34618 PMC10524887

[55] Mbulaiteye SM, Biggar RJ, Bhatia K, Linet MS, Devesa SS. Sporadic childhood Burkitt lymphoma incidence in the United States during 1992-2005. Pediatr Blood Cancer. 2009;53:366–70. 19434731 10.1002/pbc.22047 PMC2713377

[56] Guech-Ongey M, Simard EP, Anderson WF, Engels EA, Bhatia K, Devesa SS, et al. AIDS-related Burkitt lymphoma in the United States: what do age and CD4 lymphocyte patterns tell us about etiology and/or biology? Blood. 16;116:5600–4. 10.1182/blood-2010-03-275917 PMC3031406 20813897

[57] Mbulaiteye SM, Clarke CA, Morton LM, Gibson TM, Pawlish K, Weisenburger DD, et al. Burkitt lymphoma risk in U.S. solid organ transplant recipients. Am J Hematol. 2013;88:245–50. 23386365 10.1002/ajh.23385 PMC3608801

[58] Balfour HH Jr, Sifakis F, Sliman JA, Knight JA, Schmeling DO, Thomas W. Age-specific prevalence of Epstein–Barr virus infection among individuals aged 6–19 years in the United States and factors affecting its acquisition. J Infect Dis. 2013;208:1286–93. 23868878 10.1093/infdis/jit321

[59] Alaggio R, Amador C, Anagnostopoulos I, Attygalle AD, Araujo IBO, Berti E, et al. The 5th edition of the World Health Organization classification of haematolymphoid tumours: lymphoid neoplasms. Leukemia. 2022;36:1720–48. 35732829 10.1038/s41375-022-01620-2 PMC9214472

[60] Verma A, Huffman JE, Rodriguez A, Conery M, Liu M, Ho Y-L, et al. Diversity and scale: genetic architecture of 2068 traits in the VA Million Veteran Program. Science. 2024;385:eadj1182. 39024449 10.1126/science.adj1182 PMC12857194

[61] Wu B. Ferritin and iron levels inversely associated with lymphoma. Risk A Mendel Randomization Study J Hematol. 2024;13:179–85. 10.14740/jh1335 PMC11526578 39493607

[62] Stiller CA, Parkin DM. International variations in the incidence of childhood lymphomas. Paediatr Perinat Epidemiol. 1990;4:303–24. 2374749 10.1111/j.1365-3016.1990.tb00654.x

[63] Linet MS, Devesa SS. Descriptive epidemiology of childhood leukaemia. Br J Cancer. 1991;63:424–9. 2003985 10.1038/bjc.1991.98 PMC1971870

[64] Landgren O, Caporaso NE. New aspects in descriptive, etiologic, and molecular epidemiology of Hodgkin’s lymphoma. Hematol Oncol Clin North Am. 2007;21:825–40. 17908622 10.1016/j.hoc.2007.07.001

[65] Nkrumah FK. Changes in the presentation of Burkitt’s lymphoma in Ghana over a 15-year period (1969-1982). IARC Sci Publ. 1984;63**:**665–74. 6536627

[66] de-The G, Geser A, Day NE, Tukei PM, Williams EH, Beri DP, et al. Epidemiological evidence for causal relationship between Epstein-Barr virus and Burkitt’s lymphoma from Ugandan prospective study. Nature. 1978;274:756–61. 210392 10.1038/274756a0

[67] Asito AS, Piriou E, Odada PS, Fiore N, Middeldorp JM, Long C, et al. Elevated anti-Zta IgG levels and EBV viral load are associated with site of tumor presentation in endemic Burkitt’s lymphoma patients: a case control study. Infect Agent Cancer. 2010;5:13. 20667138 10.1186/1750-9378-5-13 PMC2923120

[68] Redmond LS, Ogwang MD, Kerchan P, Reynolds SJ, Tenge CN, Were PA, et al. Endemic Burkitt lymphoma: a complication of asymptomatic malaria in sub-Saharan Africa based on published literature and primary data from Uganda, Tanzania, and Kenya. Malar J. 2020;19:239. 32718346 10.1186/s12936-020-03312-7 PMC7385955

[69] Johnston WT, Mutalima N, Sun D, Emmanuel B, Bhatia K, Aka P, et al. Relationship between Plasmodium falciparum malaria prevalence, genetic diversity and endemic Burkitt lymphoma in Malawi. Sci Rep. 2014;4:3741. 24434689 10.1038/srep03741 PMC3894552

[70] Arisue N, Chagaluka G, Palacpac NMQ, Johnston WT, Mutalima N, Peprah S, et al. Assessment of mixed plasmodium falciparum sera5 infection in endemic Burkitt lymphoma: a case-control study in Malawi. Cancers*.* 2021; 13:1692. 10.3390/cancers13071692 PMC8038222 33918470

[71] Eldh M, Hammar U, Arnot D, Beck HP, Garcia A, Liljander A, et al. Multiplicity of asymptomatic plasmodium falciparum infections and risk of clinical malaria: a systematic review and pooled analysis of individual participant data. J Infect Dis. 2020;221:775–85. 31585009 10.1093/infdis/jiz510 PMC7026891

[72] Watier H, Auriault C, Capron A. Does Epstein-Barr virus infection confer selective advantage to malaria-infected children? Lancet. 1993;341:612–3. 8094840 10.1016/0140-6736(93)90364-m

[73] Chapman H, Ntemi PS, Gisiri M, Vasudevan L, Kashaigili HJ, Schroeder K. Retrospective analysis of pediatric patients with Burkitt lymphoma treated in Tanzania following the implementation of the 2016 National Treatment Guidelines: poor outcomes to current standard-of-care therapy. Pediatr Blood Cancer. 2024;71:e31145. 38924656 10.1002/pbc.31145 PMC12210323

[74] Buckle G, Maranda L, Skiles J, Ong’echa JM, Foley J, Epstein M, et al. Factors influencing survival among Kenyan children diagnosed with endemic Burkitt lymphoma between 2003 and 2011: a historical cohort study. Int J Cancer. 2016;139:1231–40. 27136063 10.1002/ijc.30170 PMC5489240

[75] Mutyaba I, Wabinga HR, Orem J, Casper C, Phipps W. Presentation and outcomes of childhood cancer patients at Uganda Cancer Institute. Glob Pediatr Health. 2019;6:2333794×19849749. 10.1177/2333794X19849749 PMC6537233 31205984

[76] Parkin DM, Youlden DR, Chitsike I, Chokunonga E, Couitchéré L, Gnahatin F, et al. Stage at diagnosis and survival by stage for the leading childhood cancers in three populations of sub-Saharan Africa. Int J Cancer. 2021;148:2685–91. 33433927 10.1002/ijc.33468

[77] Ogwang MD, Zhao W, Ayers LW, Mbulaiteye SM. Accuracy of Burkitt lymphoma diagnosis in constrained pathology settings importance to epidemiology. Arch Pathol Lab Med. 2011;135:445–50. 21466360 10.1043/2009-0443-EP.1 PMC3357109

