# Supplementary material for: A genome-wide association study identifies an African-specific locus on chromosome 21q22.12 associated with Burkitt lymphoma risk and survival
Source: Leukemia. 2025 Jul 11;39(9):2196–206. doi: 10.1038/s41375-025-02690-8 (PMC12380610; doi:10.1038/s41375-025-02690-8)
Supplement: Supplementary file 1 — Supplementary Material [file 41375_2025_2690_MOESM1_ESM.docx]

Supplementary Information

**A genome-wide association study identifies an African-specific locus on chromosome 21q22.12 associated with Burkitt lymphoma risk and survival**

Dutta *et al***.**

**Supplementary Figures**

[Section 1 - Methods 3](#_Toc197430958)

[1.0. Study populations, enrollment, and ethics 3](#_Toc197430959)

[2.0. DNA extraction, genotyping, and quality control 5](#_Toc197430960)

[3.0. Population structure and relatedness. 5](#_Toc197430961)

[3.1. GWAS analysis 6](#_Toc197430962)

[3.2. Fine mapping to identify causal variants 7](#_Toc197430963)

[3.3. Sensitivity analyses 7](#_Toc197430964)

[4,0. Genomic and transcriptomic analyses in the BLGSP 8](#_Toc197430965)

[4.1. eQTL analysis 9](#_Toc197430966)

[4.2. Long-read whole-genome sequencing of EB-3 cell line 9](#_Toc197430967)

[4.3. Investigation of potential regulatory activity of the chr21q22.12 CS-SNPs 10](#_Toc197430968)

[4.4. Prediction of transcription factor-binding sites 11](#_Toc197430969)

[4.5. Analysis of DNA-protein interactions 11](#_Toc197430970)

[4.6. Analysis of long-range chromatin interactions 12](#_Toc197430971)

[5.0. Retrospective follow-up of BL patients and survival analysis 13](#_Toc197430972)

[6.0. Gene aggregated multi-marker analyses 14](#_Toc197430973)

[7.0. Data availability. 14](#_Toc197430974)

[Section 2 – Supplementary Figures 15](#_Toc197430975)

[Fig. S1. Basic design of the BL GWAS. 15](#_Toc197430976)

[Fig. S2. Population substructure among participants in the BL GWAS. 16](#_Toc197430977)

[Fig. S3. Population-specific principal component analysis (PCA) in the BL GWAS. 17](#_Toc197430978)

[Fig. S5. Characteristics of participants in the BL GWAS. 19](#_Toc197430979)

[Fig. S6. Genomic inflation plot using the combined dataset. 21](#_Toc197430980)

[Fig. S7. Association of the top BL GWAS lead rs111457485-T stratified by country and comparison with main/original analysis. 22](#_Toc197430981)

[Fig. S8. The chr21q22.12 genomic region flanked by the RUNX1 and SETD4 genes. 23](#_Toc197430982)

[Fig. S10. Long-read RNA-sequencing in a B-cell diffuse large cell lymphoma (DLBCL) cell line OCI-LY7. 25](#_Toc197430983)

[Fig. S11. Analysis of the regulatory activity of the chr21q22.12 CS-SNPs by Luciferase reporter assays. 26](#_Toc197430984)

[Fig. S12. The evaluation of DNA‒protein interactions of regulatory chr21q22.12 CS-SNPs by electrophoretic mobility shift assays (EMSA). 27](#_Toc197430985)

[Fig. S13. The genomic landscape of the chr21q22.12 BL GWAS locus. 29](#_Toc197430986)

[Fig. S14. Flow chart showing retrospective follow-up of 249 Ugandan BL patients to determine vital status and chemotherapy information. 30](#_Toc197430987)

[Fig. S15. Overall survival (OS) of 228 Ugandan patients after BL diagnosis according to select characteristics. 31](#_Toc197430988)

[Fig. S16. A suggestive 3q26.1 GWAS locus tagged by the SNP rs9847876. 32](#_Toc197430989)

[Fig. S17. Additional BL-GWAS signals within the 19p13.2 locus identified by multi-marker tests. 33](#_Toc197430990)

[Fig. S18. Comparative analysis of the rs2242780 genomic region. 34](#_Toc197430991)

[Fig. S19. A proposed model to explain the associations for rs111457485-T and rs2242780-C with BL risk and survival. 35](#_Toc197430992)

[Fig. S20. Evaluation of batch effects in Uganda genotype results. 36](#_Toc197430993)

[Fig. S21. Principal Component Analysis to assess substructure of BL cases analyzed by select characteristics. 37](#_Toc197430994)

[Fig. S22. Distribution of anatomic sites in 512 BL patients from the EMBLEM study with data, by enrollment year. 38](#_Toc197430995)

[Section -3 References 39](#_Toc197430996)

# Section 1 - Methods

## 1.0. Study populations, enrollment, and ethics

The BL GWAS was conducted in participants enrolled in the EMBLEM study in Uganda, Tanzania, and Kenya^1^ and the Childhood Infections and Cancer study in Malawi^2^. Briefly, the EMBLEM study enrolled population-based cases and controls aged 0-15 years from six neighboring rural geographical areas—two each in Uganda, Kenya, and Tanzania (**Figure 1a**) during 2010-2016. The cases were children with a BL diagnosis based on local histology or cytology (available on 74% of cases^3^) and only clinical evidence in the remainder enrolled from six hospitals in the study area. A small number of Ugandan BL cases (*n*=38) were also enrolled in the Burkitt Lymphoma Genome Sequencing Project (BLGSP)^4^, providing us a set of cases that were rigorously evaluated and also had tumor whom genome sequencing (WGS) and RNA-Sequencing (RNA-Seq) data for transcriptomic analyses. The BLGSP cases from EMBLEM were similar with respect to demographic and clinical features to the EMBLEM cases who were not enrolled in the BLGSP (**Table S14**).

The EMBLEM controls were healthy children with no prior history of BL or any other cancer enrolled at home from 295 study villages (100 in Uganda, 100 in Kenya, and 95 in Tanzania) randomly selected from all villages in the six regions^1^. Demographic and clinical risk factors for BL were collected from the cases and controls using structured interviewer-administered questionnaires^1^. The anatomic site of BL is a correlate for the stage^5^ and severity of BL^6, 7^. It was ascertained using anatomic site forms (<https://emblem.cancer.gov/resources/forms_case/Tumor_Anatomic_Site-HIPE2.pdf>) and classified based on the Toronto consensus principles and guidelines for cancer-specific staging for cancer registries^8^.

Venous blood samples were collected from both cases and controls and immediately tested for *P. falciparum* infection using thick-film blood smear microscopy for presence of asexual forms and antigen capture rapid diagnostic tests (RDTs) presence of antigenemia, and for HIV infection using serology^1^. Research samples were separated into plasma, buffy coat and red cell fractions for storage at −80°C until testing. HIV infection was rare (24 cases and 15 controls were positive^1^), consistent with the low HIV prevalence in children and the weak association of HIV with BL in Africa^9^. As HIV status did not alter the associations of BL with the sickle cell trait^3^, HLA^10^, or demographic characteristics^1^, HIV-positive participants were not excluded from the GWAS.

The Malawi study enrolled participants aged 0-17 attending the Queen Elizabeth Hospital in Blantyre for cancer treatment ^2^ during 2005-2008. Caes from the whole country were eligible (**Figure 1a**). The cases were children diagnosed with BL, based on local histology, cytology, or only clinical diagnosis, and the controls were children with other non-lymphoid solid malignancies. Venous blood was collected in EDTA tubes before treatment and stored in -20°C freezers prior to transfer to the US National Cancer Institute (NCI). Participants with HIV or Kaposi sarcoma were excluded prior to sharing data for ethical reasons (confidentiality and privacy). *P. falciparum* infection in Malawi was detected via *P. falciparum*-specific PCR^11^.

We confirm that all relevant ethical regulations were followed. The approval for the EMBLEM study was granted by ethics committees at the Uganda Virus Research Institute (UVRI, GC/127), the Uganda National Council for Science and Technology (HS 816), the Tanzania National Institute for Medical Research (NIMR/HQ/R.8c/Vol. IX/1023), Moi University/Moi Teaching and Referral Hospital (000536), and the US National Cancer Institute (10-C-N133). Permission to retrospectively recontact patients to determine the vital status of all 249 Ugandan BL patients included in the GWAS was obtained from UVRI (GC/127/178). The original ethical approval for the Infections and Childhood Cancer Study in Malawi was granted by ethics committees at the Malawi College of Medicine (P.03/04/277R) and Oxford University. Permission to conduct genetic testing on residual samples from Malawi was obtained from the Malawi National Health Sciences Research Committee in 2019 (Approval #2405). Written informed consent was obtained from the participants’ guardians in the EMBLEM and Malawi studies, and written informed consent was obtained from children aged ≥7 years in the EMBLEM study. Ethical approval for GWAS was granted on the stipulation that research focused on the established risk factors for BL, including malaria, malaria resistance genes^3^ and *HLA* variation^10^, was prioritized.

## 2.0. DNA extraction, genotyping, and quality control

DNA extraction and genotyping of all samples were performed at the Cancer Genomics Research (CGR) Laboratory, NCI, USA. The laboratory staff were blind to the case-control status of the samples. The genotypes of approximately 4.6 million variants were determined using the Infinium Omni5Exome-4 v1.3 BeadChip (Illumina) following the standard Illumina data analysis workflow^3, 10, 12, 13^. The samples from Uganda were run on three separate runs (batches), while those from Tanzania, Kenya, and Malawi were run on one batch only. Quality control (QC) of the genotype data, including filtering based on SNP/sample call rate, assay concordance, genetic sex prediction based on the X chromosome markers, and sample contamination rates, was performed using standard CGR pipelines^3, 13^. Principal component analysis (PCA)^14^ was conducted to identify batch effects (**Fig. S20a**), which were mitigated by removing SNPs (*n*=27,039) with extreme PCA weights across the three genotype batches (**Fig. S20b).** PLINK 1.9^14^ was used for QC filtering minor alleles(MAF <1%) per-variant and per-individual missing data (<5%), and variants deviating from Hardy‒Weinberg equilibrium in the controls (*p* < 1×10^‒6^). The 2,267,535 high-quality SNPs were phased and imputed with EAGLE2^15^ and Positional Burrows-Wheeler Transform (PBWT)^16^ using the African Genome Resources (AGR) as a reference panel ([imputation.sanger.ac.uk](https://imputation.sanger.ac.uk/)). The AGR was preferred because it had the largest number of genomes from African individuals from the Great Lakes region (*n*=6,230), which overlaps substantially with the BL belt covered by the current study^12^. Imputation resulted in a dataset of ~ 26 million high-quality genotyped or imputed SNPs. Variants with a MAF > 0.01, imputation INFO score > 0.3 and minor allele count >20 (*n* ~ 22 million) were retained for the GWAS analyses.

### 3.0. Population structure and relatedness.

PLINK 1.9^14^ was used to prune the 2,267,535 high-quality genotyped SNPs using an LD threshold of 0.3 in a sliding window of 50 bp and a shift step of 10 bp to obtain 727,834 independent (*r^2^* < 0.3) SNPs. Principal component analysis (PCA)^14^ was performed based on the 787,731 independent SNPs to assess population structure in the combined set (**Fig. S2**) and separately for each country to calculate population-specific principal components (PCs, **Fig. S3**). The top 3 population-specific PCs were used as covariates for genomic control. The independent SNPs were used to construct a pairwise genetic relatedness matrix (GRM) of all study participants based on the probability that two individuals *i* and *j* share 0, 1, or 2 alleles using the identical by descent (IBD) (δ^0^_𝑖𝑗,_ δ^1^_𝑖𝑗,_ and δ^2^_𝑖𝑗_, respectively)^17^. A pairwise kinship coefficient (*Φ_ij_*) for each participant was calculated as a function of IBD-sharing, *Φ_ij_* = *1/2*δ^2^_𝑖𝑗_ + *1/4*δ^1^_𝑖𝑗,_ based on the estimated IBD probabilities.

The population structure between EMBLEM BL cases enrolled in the BLGSP versus those not enrolled was evaluated (**Fig. S21a**) using 393,851 independent SNPs from the exome sequencing data, following the same filtering steps used for the genotype data. We observed some differences in PCA patterns between BLGSP patients from EMBLEM versus those enrolled from Kampala in Uganda, suggesting variability in the geographical areas or ancestral groups of origin and possibly referral patterns of cases enrolled in northern Uganda versus those enrolled in Kampala in Uganda (**Fig. S21b**). In EMBLEM, PCA plots did not identify differences suggesting that anatomic site of BL and *P. falciparum* positivity are evenly distributed in the geographical and ancestral space of EMBLEM participants (**Fig. S21c and d**).

## 3.1. GWAS analysis

GWAS with ~ 22 million loci with INFO >0.3 and minor allele count >20 was performed in the combined dataset by fitting logistic mixed model regression performed in SAIGE^18^ (version 1.1.6.2), including age, genetic sex, country, three top population-specific PCs (ps-PCs) as fixed-effects covariates and the GRM as a random-effect.

BL case_status ~ SNP + age + sex + country + ps-PC1 + ps-PC2 + ps-PC3 + [GRM]

Age was fit as a continuous variable in single years. Genetically inferred sex was used for 31 individuals whose clinical records were discordant due to clerical errors^13^. Leave-one-chromosome-out model fitting was enabled with approximate Firth effect size estimation^19^ for SNPs with *P* < 0.05. The used of GRM to account for relatedness between participants allowed keeping related individuals in the dataset and therefore preserving a sample size. A genome-wide significance threshold of *P*<5×10^−08^ was considered statistically significant, whereas results with *P*<5×10^-7^ but *P*>5×10^−08^ were considered suggestive.

### 3.2. Fine mapping to identify causal variants

Fine mapping was performed using Sum of Single Effects (SuSiE) model^20^ to identify a set of SNPs with > 95% probability of including the causal variant(s) for the GWAS locus, hereafter referred to as the credible set SNPs (CS-SNPs). The ancestral patterns of the CS-SNPs were evaluated in the long-read whole-genome sequencing (WGS) of the Human Pangenome Reference Consortium (HPRC)^21^ and in archaic humans as described elsewhere^22^.

### 3.3. Sensitivity analyses

Several sensitivity analyses were performed. The GWAS results from the combined set were compared to GWAS performed using a standard meta-analysis of the country-specific ORs, with the heterogeneity in the country-specific ORs evaluated using Cochran’s Q test. The -log10(p-value) of the significant associations in original (combined set) analysis versus –log10(p-value) of the results based on meta-analysis were compared to check for concordance between the results from either analysis **(Fig. S7**). To verify that the adjustment for genetic relatedness was adequate, the GWAS was repeated after removing PC outlier individuals (*n*=346, **Fig. S4**), defined as having population-specific PCs >1.5 * interquartile range, and additionally removing first- or second-degree relatives (n=609), based on KING to generate a maximal independent set, and the results compared to those generated with the full cohort (**Table S2**). Finally, additional adjustment for factors associated with BL was done, namely, *P. falciparum* infection^1^, plasma EBV DNA detection, EBV antibody titers (**Table S3**), the *HBB*-rs334-T allele^3^, rs2040406-G in the *HLA-DQA1* region^10^, and age, stratified as < or >=9 years, which is correlated peak exposure to malaria and naturally acquired immunity to malaria^23^, and tumor anatomic site.

Tumor anatomic sites were grouped into four categories: head-only (*n*=177), abdominal-only (*n*=253), head and abdominal (*n*=65), and other/disseminated (*n*=18) based on information collected at baseline following the Toronto consensus principles and guidelines for cancer-specific staging systems for use by registries^8^. Head-only tumors characterize a limited disease, while abdominal tumors characterize advanced disease. Although a previous report suggested that the proportion of abdominal BL rose with calendar time ^24^, an apparent increase was only noted for head-only BL (**Fig. S21**).

Circulating EBV DNA in plasma results are based on subsets of EMBLEM subjects in whom these makers have been measured using EBV digital droplet PCR targeting the 71 bp BamHI–W internal repeat regions of EBV. The EBV antibody results are based on the EBV proteome away measurement conducted in EMBLEM participants using results for three peptides (BHRF1: YP_001129442.1-42204-42779; and two peptides of BMRF1: YP_001129454.1-67745-68959, AFY97929.1-67486-68700) previously identified as strongly associated with BL in Ghana^25^ (manuscripts under preparation).

4,0. Genomic and transcriptomic analyses in the BLGSP

As genomic and transcriptomic datasets from African-ancestry individuals are limited, WGS and RNA-seq data from the BLGSP^4^ were accessed through the NCI Genomic Data Commons portal (GDC, [https://portal](https://portal/).gdc.cancer.gov/; Project ID: CGCI-BLGSP, accession: phs000527.v13.p4). Controlled access Bam files were downloaded for DNA-WGS and RNA-seq for tumor and matching blood samples (all for short-read Illumina sequencing). Precalculated total RNA-seq expression values were downloaded as transcripts per million (TPMs). Transcriptome-aligned RNA-seq bam files were used to quantify isoform-level expression with Rsem^26^. BAM files were used to review regions of interest with Integrative Genomic Viewer (IGV, version 2.16.1). Genotypes of genetic variants of interest were directly scored from DNA-WGS bam files using igvtools count (v2.12.3).

The associations of total and isoform-level expression with BL-associated CS-SNPs were evaluated using linear regression models unadjusted or adjusted for age, sex, and tumor EBV status (positive/negative). The tumor mutational burden (TMB) was defined as the sum of tumor-specific single base substitutions and small (<50 base pairs) insertions/deletions in coding and non-coding regions present in tumor but not the matching paired normal sample^27^. Four mutational COSMIC signatures previously identified in BLGSP BL tumors^27^ were analyzed: signature A (COSMIC age-associated, signature SBS5), signature B (COSMIC signature SBS17 with unknown etiology), signature C (COSMIC signature SBS15 associated with defective DNA mismatch repair), and signature D (COSMIC signature SBS9 associated with AICDA and polymerase η activity).

### 4.1. eQTL analysis

Cis-acting expression quantitative trait loci (eQTLs) were explored by querying BL GWAS variants in existing transcriptomic databases, including eQTLGen (whole blood, *n*=31,355), GTEx (*n*=54 tissues from 838 donors), MuTHER lymphoblastoid cell lines (LCLs, *n*=856)^28^, and the Jackson Heart Study (JHS) of controls of African ancestry (PBMCs, *n*=1012)^29^.

### 4.2. Long-read whole-genome sequencing of EB-3 cell line

DNA samples from BL-derived cell lines, some with corresponding paired lymphoblastoid cell lines (LCLs) from the same patient (**Table S15**) obtained from the Deutsche Sammlung von Mikroorganismen und Zellkulturen cell line bank (courtesy of Dr. Hilmar Quentmeier) or under the framework of the MMML Network from Michael Hummel and Harald Stein (Institute for Pathology, Charite, Berlin) were genotyped with the Multi-Ethnic Global Illumina genotyping array by the CGR Laboratory, NCI, using a standard Illumina data analysis workflow. Only one cell line (EB-3, derived from BL in a Ugandan child^30, 31^) carried the effect alleles of chr21q22.12 SNPs. DNA was extracted from 5–10 million EB-3 cells using the Qiacube (Qiagen), and 1 µg of DNA was sequenced on PromethION flow cells (Oxford Nanopore) with washing and reloading 3 times, generating a total of ~15.5 million raw long reads (109 GB). The reads were aligned to the hg38 assembly of the human genome using a Nanopore alignment pipeline of the Cancer Genomics Cloud.

### 4.3. Investigation of potential regulatory activity of the chr21q22.12 CS-SNPs

The cell lines EB3, Raji, DG-75, and HEK293T were purchased from ATCC (Manassas), grown in suggested conditions and verified by Identifiler if used for over one year after purchase. These cell lines were used in the *in vitro* experiments were grown under the recommended conditions and regularly tested for Mycoplasma contamination using the MycoAlert Mycoplasma Detection Kit (Lonza). The sequences flanking the SNPs (~ 250 bp on each side) were cloned into a Luciferase reporter vector with a basic promoter (pGL4.23, Promega). The fragments were ordered as custom allele-specific plasmids (Integrated DNA Technologies) in which target sequences were flanked by recognition sites for the KpnI restriction enzyme. The pGL4.23 vector and source allele-specific plasmids were digested with the KpnI restriction enzyme (New England Biolabs), ligated, and transformed into One Shot TOP10 competent *E. coli* cells (Thermo Fisher). The constructs were confirmed by Sanger sequencing to select 4 constructs per variant (with allele-specific inserts cloned in both the forward and reverse orientations). The selected plasmids were purified with an Endotoxin-free Plasmid Maxi Kit (Qiagen).

Each plasmid was first tested in HEK293T cells (normal embryonic kidney cells) with high transfection efficiency (48.6% for the control pmaxGFP plasmid, Lonza). The cells were seeded in 96-well plates (10^5^ cells per well) and transiently transfected 24 h later with 100 ng of a plasmid using Lipofectamine 3000 transfection reagent (Thermo Fisher). On each plate, the cells were similarly transfected with the pGL4.13 plasmid [luc2/SV40, Promega], which was used as a positive control, and the empty pGL4.23 plasmid, which was used as a negative control. All the transfections were performed with 6–12 technical replicates.

Luciferase activity was measured 24 hours post-transfection. The cells were gently rinsed with PBS and lysed in the same wells with 20 µl of Passive Lysis Buffer per well, and 10 µl of cell lysate was transferred onto a white flat-bottom 96-well plate and assayed for Renilla and Firefly Luciferase activity using Dual-Luciferase Reporter Assay Reagent (E1960) and GloMax Explorer (Promega).

The variants with putative enhancer activity identified in HEK293T cells were then tested in a BL-derived cell line (Raji). Raji cells were transfected using 4D-Nucleofector X Unit (Lonza) using the SG Cell Line 4D-Nucleofector X Kit S (Lonza, V4XC-3032) and the preset program DS-104, achieving ~25% transfection efficiency for a control pmaxGFP plasmid. The cells were transfected with 600 ng of plasmids per 4x10^5^ cells in 6–12 technical replicates. Luciferase activity was measured 24 h post-transfection via the same method used for the HEK293T cells. For both cell lines, transfections were performed in 2–3 biological replicates on different days, and the results from one representative experiment are presented. The results from the GloMax Explorer were analyzed by normalizing the Luciferase luminescence to that of the Renilla endogenous control and the pGL4.23 negative control. The fold differences for the minor alleles compared with the major allele (used as a reference) were further analyzed and plotted with Prism 10 (GraphPad); p values are from two-sided unpaired t-tests. The results are plotted as individual values and group means with error corresponding to the standard deviations.

### 4.4. Prediction of transcription factor-binding sites

Potential regulatory effects of the 17 chr21q22.12 CS-SNPs were assessed via *in silico* prediction of transcription factor-binding sites (TFBS) through the following online tools: <https://opera.autosome.org/perfectosape/scan> and TRAP 3.05 (<https://trap.molgen.mpg.de/cgi-bin/trap_two_seq_form.cgi>). Allele-specific sequences for each SNP were queried for potential significant differences in binding and fold change.

### 4.5. Analysis of DNA-protein interactions

SNPs with enhancer regulatory activities identified through the Luciferase Reporter Assays were further tested for DNA-protein interactions by electrophoretic mobility shift assays (EMSAs). Oligonucleotides encompassing the variants with 10 base pairs of flanking sequences on each side (21 bp in total) were synthesized and purchased as unlabeled or labeled at the 5’ end with IRDye 700 (Integrated DNA Technologies). Complementary oligonucleotides were annealed at 100°C to form double-stranded DNA probes.

EMSAs were carried out using a LightShift EMSA Optimization and Control Kit (Thermo Fisher) using nuclear extracts from 293T, Raji, and EB3 cells prepared with the NE-PER Nuclear and Cytoplasmic Extraction Reagents (Thermo Fisher). Binding reactions included the nuclear extract (8 µg), buffer and either the labeled probe alone (no competition), the labeled probe mixed with an ×100 excess of unlabeled self-probe (specific competition), or the probe with the opposite allele of the SNP (nonspecific competition). Electrophoresis was performed on a 4–20% Tris-borate-EDTA (TBE) gradient gel under nondenaturing conditions on ice. After electrophoresis, the gel was imaged using an IRDye680RD Blot on a ChemiDoc Imaging System (Bio-Rad Laboratories). Allele-specific binding was determined by comparing the band shifts and intensities between lanes corresponding to different alleles and conditions.

### 4.6. Analysis of long-range chromatin interactions

Pore-C libraries for BL-derived EB-3, Raji, and DG-75 cell lines were prepared following Oxford Nanopore Technology protocols (<https://nanoporetech.com/document/telomere-to-telomere-sequencing-t2t-on-promethion-sqk-apk114-sqk)(https://nanoporetech.com/document/restriction-enzyme-pore-c>). Briefly, 10 million fresh cells were crosslinked with 1% formaldehyde for 10 minutes at room temperature, and crosslinking was stopped with 1% w/v glycine. A total of 5 million crosslinked cells underwent permeabilization, ligation, and denaturation, yielding approximately 15 μg of DNA after precipitation. A total of 1 µg of each DNA sample was used for DNA library preparation using the Oxford Nanopore reagents Ligation Sequencing Kit V14 (SQK-LSK114), with washing with the Long Fragment Buffer to exclude smaller DNA fragments. The library was loaded onto Oxford Nanopore PromethION flow cells for sequencing, followed by washing and reloading 1–2 times. The raw Nanopore sequencing files were converted to FASTQ format using Guppy (v6.5.7) and processed through the Pore-C workflow (https://github.com/epi2me-labs/wf-pore-c) to generate Hi-C files. For visualization, Hi-C files were converted to .cool format using HiCExplorer (v3.7.2) and then to BEDPE format using HiCCompare (v3.19) in R. Chromatin interactions were filtered based on locations of 17 CS-SNPs and their interaction frequencies and uploaded to the UCSC Genome Browser. All analyses were performed using the NIH HPC Biowulf cluster (https://hpc.nih.gov). The ChiP-seq tracks of EBNA2, EBNA2, and EBNA3 were downloaded from the NCBI SRA (GSE47629 and GSE73887) and uploaded to the UCSC genome browser as custom tracks.

## 5.0. Retrospective follow-up of BL patients and survival analysis

Limited clinical follow-up was performed for the 249 BL GWAS cases from Uganda enrolled during 2010--2016 (**Figure 1b**). After ethics approval in Uganda, Community Research Assistants (local health workers and EMBLEM field staff) recontacted by phone or home visits patients and their parents/guardians to invite them to participate in the follow-up component. Self-consent was provided by patients who were now adults (>18 years) and emancipated minors defined as individuals aged 14-18 years (n=91) who were living independently or were married, while parental/guardian consent was obtained from patients who were still minors (<14 years n=29). After providing consent, respondents answered a brief questionnaire about the vital status of the BL patient. Patient files were retrieved from the hospital records to abstract treatment information. The study data were managed using the REDCap electronic data capture tools hosted at the Infectious Diseases Institute at Makerere University College of Health Sciences (Uganda), and only deidentified data were shared with the NCI.

Of the 249 Ugandan EMBLEM patients, the data were analyzed for 228 BL patients whose vital status information (106 deaths) was ascertained (**Fig. S14a; Table S10**). The 21 cases whose vital status information was not obtained were not different by sex or age from those whose who were traced. Overall survival (OS) was estimated via univariable or multivariable Cox proportional hazard regression models for all 228 patients overall and stratified by tumor anatomic site as a significant clinical factor. OS was analyzed using the log-rank test, and Kaplan‒Meier plots were generated using the R package survminer (version 0.4.9). Associations of specific SNPs with OS were explored using Cox proportional hazard regression models, as implemented in the R package survival (version 3.6-4), adjusted for age, sex, plasma EBV status, number of chemotherapy drugs administered (0 for untreated and 1-6 drugs), and tumor anatomic site (head-only, abdominal-only, head and abdominal and other). Further analyses adjusted for the same covariables were stratified by tumor anatomic site (**Fig. S14b, Fig. S15)**.

## 6.0. Gene aggregated multi-marker analyses

Multi-marker association testing for gene effects across the genome was performed using the MAGMA package (version 1.08)^32^. MAGMA uses multiple regression to aggregate variants that reside in the +/- 20 kb neighborhood of a gene body as defined by the Refseq database to incorporate potential regulatory variants. A transcriptome-wide association study (TWAS) was performed using FUSION (https://gusevlab.org/projects/fusion/) with the trained GTEx v8 models, using the LD SNPs from the individuals of African ancestry from the 1000G. TWAS analyses excluded the human leukocyte antigen (HLA) region because of its complex LD patterns. The significance threshold of TWAS associations was determined using a per-tissue Bonferroni threshold, correcting for the number of gene-models in each tissue type.

## 7.0. Data availability.

The genetic data reported in this paper are available through dbGaP under accession code phs001705.v1.p1 (EMBLEM), and the BLGSP files can be accessed from the Genomic Data Commons (GDC, <https://portal.gdc.cancer.gov/>; Project ID: CGCI-BLGSP, dbGaP study accession: phs000527.v13.p4). The Pore-C and whole-genome sequencing data for BL cell lines generated in this study have been deposited in the NCBI with a BioProject accession number (RJNA1212064; <https://dataview.ncbi.nlm.nih.gov/object/PRJNA1212064?reviewer=rcom8aun4bsimui03hb40bo0i>). The Million Veteran Program (MVP) data for African American participants was accessed at <https://phenomics.va.ornl.gov/pheweb/gia/afr/variant/21:35703388-G-C> for rs2242780 and <https://phenomics.va.ornl.gov/pheweb/gia/afr/variant/21:35702601-C-T> for rs111457485.

Covariate data for EMBLEM can be applied for directly by request to the corresponding author (SMM).

# Section 2 – Supplementary Figures

##
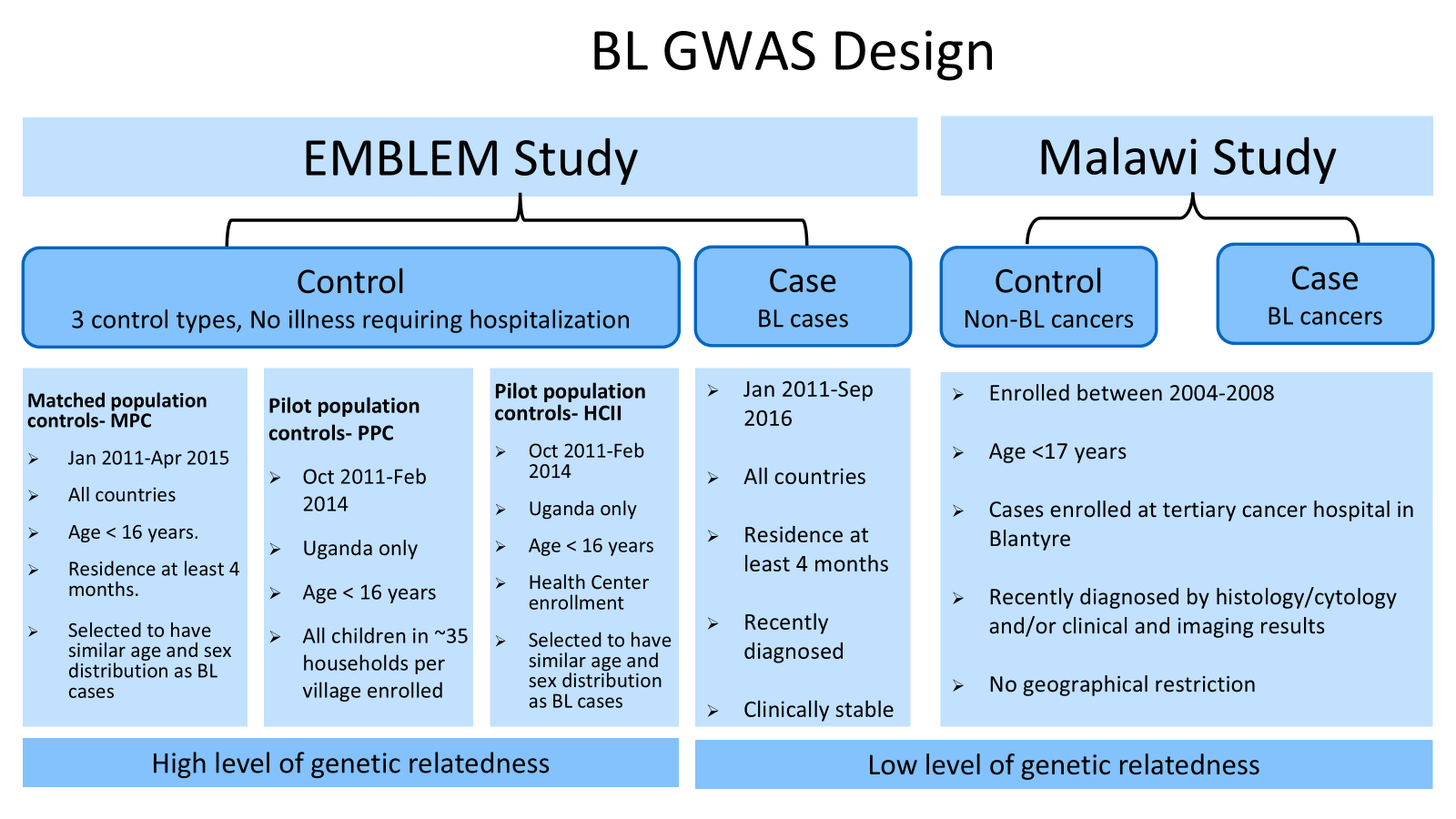
Fig. S1. Basic design of the BL GWAS.

The participants in the EMBLEM study were enrolled from six geographically defined regions (Figure 1a) as population-based BL patients and three sets of controls: a) matched population controls enrolled from their homes in 300 villages in Uganda, Tanzania, and Kenya^1^; b) pilot population controls enrolled via a household survey design in 12 pilot villages in Uganda^33^; and c) health center II controls enrolled from children attending local health center units in 12 pilot villages for minor ailments in Uganda only^34^. The participants in Malawi were enrolled in a tertiary cancer care hospital in Blantyre, Malawi. The cases were children diagnosed with BL, and the controls were children with other solid cancers^35^.

## Fig. S2. Population substructure among participants in the BL GWAS.


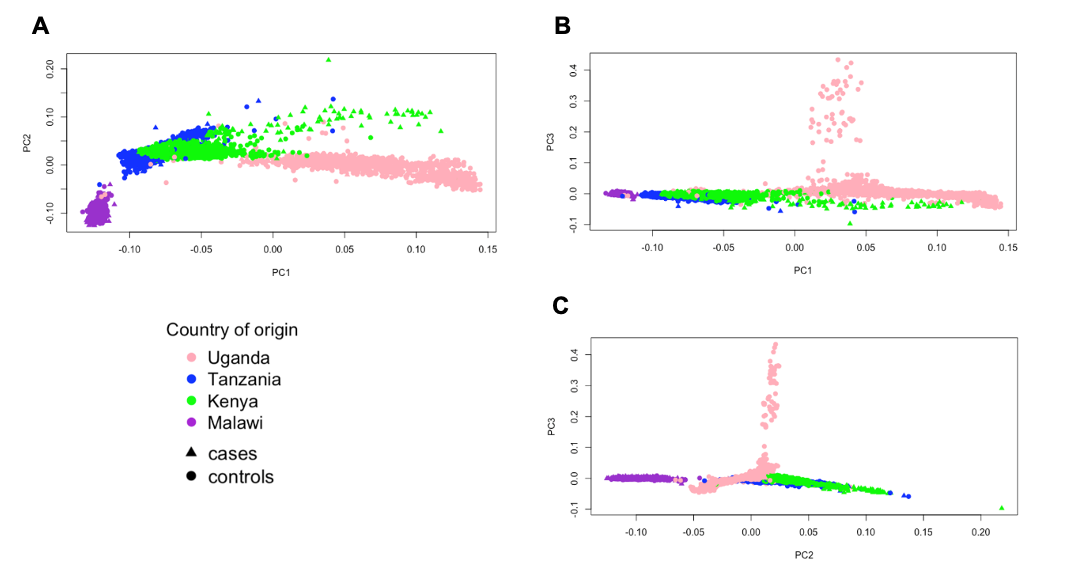


Principal components (PCs) (based on in-cohort analysis of all population combined and 727,834 uncorrelated SNPs). Participants from the different countries are shown using different colors (pink-Uganda, blue–Tanzania, green–Kenya, and purple-Malawi). The case status of the participant is represented by solid triangle-cases, solid circle-controls. Top PCs: a, PC1 versus PC2; b, PC1 versus PC3; cPC2 versus PC3. PCs for BL cases and controls cluster together in each country, suggesting there is no population structure between the BL patients and controls in all the countries, as previously reported^3, 10^. PC3 captures relatedness among Ugandan controls that was adjusted for in the GWAS models using the genetic relationship matrix.

## Fig. S3. Population-specific principal component analysis (PCA) in the BL GWAS.


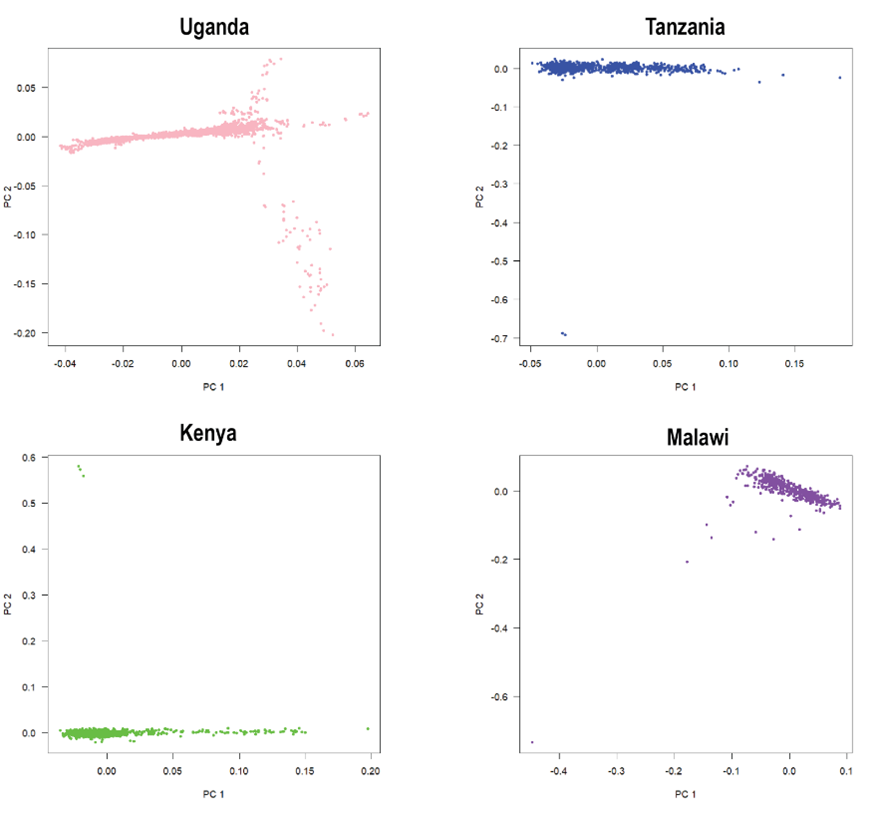


The Plots of population-specific PCs based on PCA performed separately within each population (country) based on 727,834 uncorrelated SNPs variants (removing ATCG SNPs, --geno 0.05, --hwe 10^--6^, r^2^ < 0.2). The top 3 population-specific PCs were incorporated in the GWAS model.

Fig. S4. Population-specific principal component analysis in the BL GWAS showing PC outliers by country.


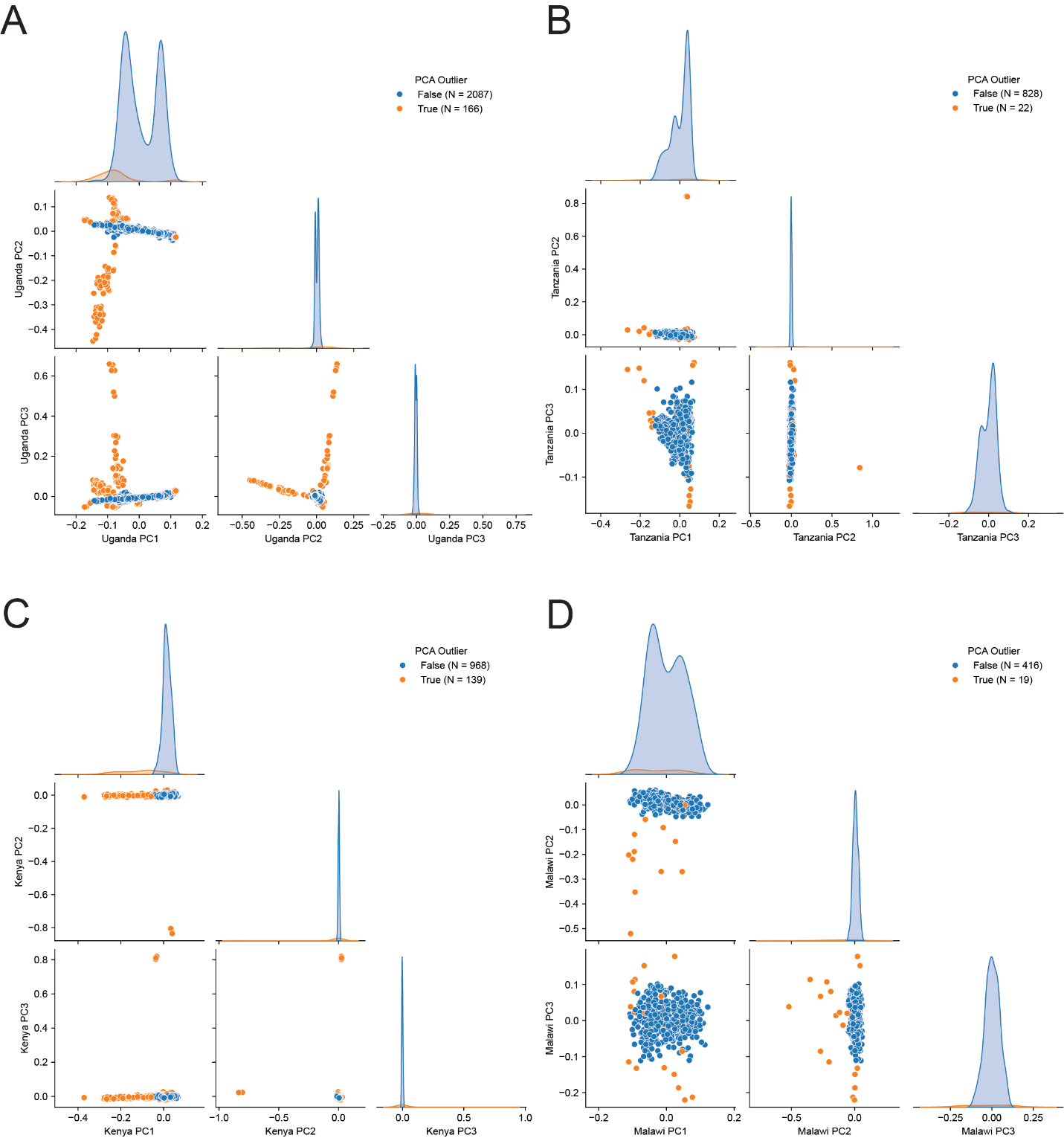


The outliers, defined as samples with population-specific PCs greater than 1.5 * interquartile range (IQR), and related individual based on KING to define the maximal independent set without any first- or second-degree relatives, were removed from the sample in sensitivity analyses to confirm that the main models adequately handled relatedness in the sample.

## Fig. S5. Characteristics of participants in the BL GWAS.


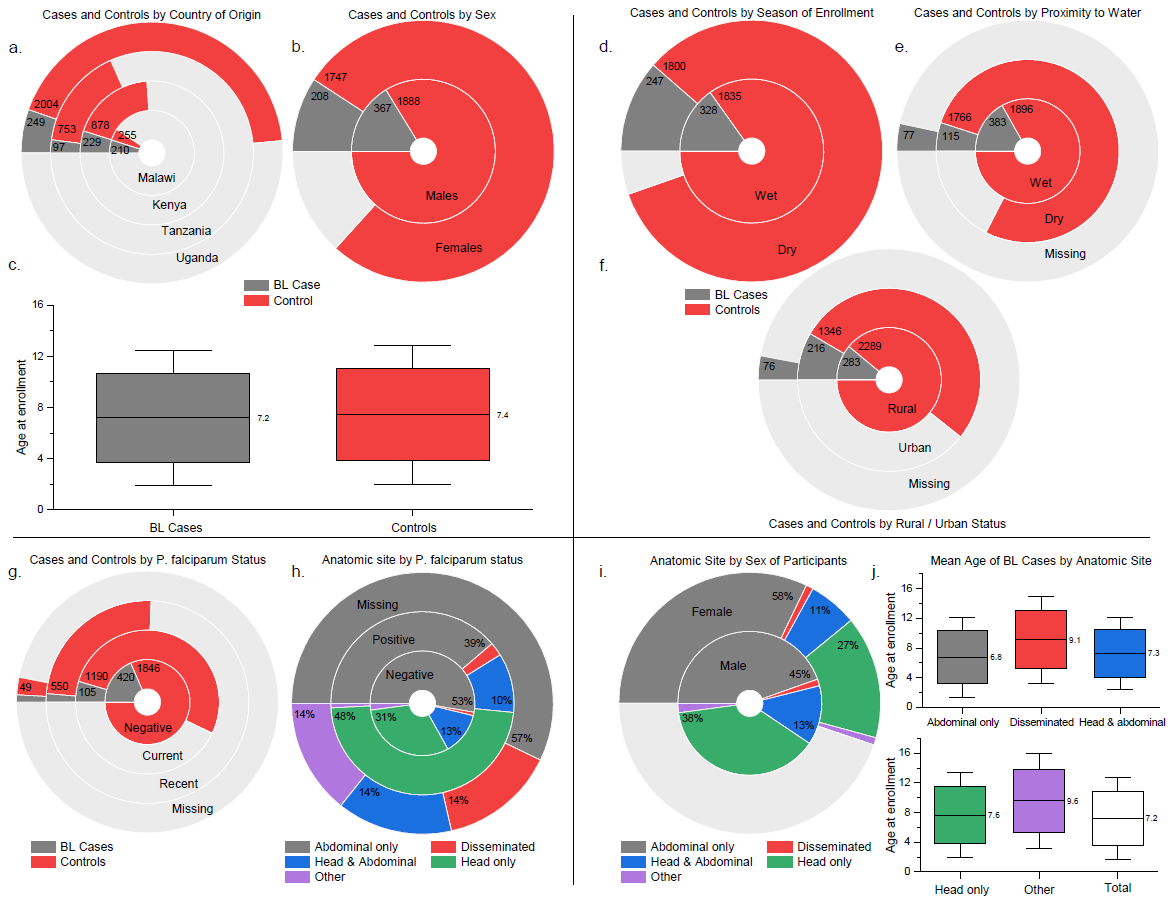


The demographic characteristics of the BL GWAS cohort have previously been reported^1, 3^. A summary of the patterns is in panel a-c are for all GWAS participants (*n*=4,645), panels d-h are based on EMBLEM participants with detailed geospatial data (*n*=4210), and panels i-j are based on EMBLEM participants with anatomic site data (*n*=512). The results are not statistically significant unless when indicated in the legend. Distribution of BL patients and controls by: a, country of enrollment; b, sex showing predominance of girls (*P*<0.0001); c, the mean age at enrollment showing the cases to be slightly younger than the controls (*P*=0.02). d, the season of enrollment showing that BL cases were more likely to be enrolled during the wet season (*P*=0.003). e, proximity of the participant’s village to surface water (lake, river, or swamp) (<500 m (wet) or >500 m (dry) showing that BL patients are more likely to be enrolled from villages near water (*P*<0.0001, based on those with nonmissing values). f, urban/rural status of the participant’s village showing that BL patients were more likely to be enrolled from rural villages (*P*=0.007, based on those with nonmissing values). Urban status was defined based on a census population count above the average of all villages in the region; otherwise, rural status. g, Current or recent *P. falciparum* infection status was associated with a lower frequency of infection in BL patients than in controls (18,9%/5.4% versus 33.2%/15.3%, *P*<0.0001, based on those with nonmissing values). The results are based on thick-film microscopy (current infection, when positive) and rapid diagnostic tests (recent infection when positive but negative on thick-film microscopy). The participants who were negative for both tests were considered negative. h, BL tumor anatomic site involvement by *P. falciparum* infection status, defined as positive or negative, showed that *P. falciparum* infection was more frequently detected in cases with head-only than in those with abdominal-only BL (47.6% versus 38.7%, respectively; Fisher’s exact *P*=0.001). i, BL tumor anatomic site by sex, with head-only BL predominating in males and abdominal BL predominating in females (72% versus 58%, Fisher’s exact *P*=0.05). j, The mean age at diagnosis of cases with BL tumors involving different anatomic sites showed slight differences in age (ANOVA *P*=0.014). The median lines show the means, and the two whiskers for each box plot mark the minima and maxima based on a standard deviation of 1.5.

## Fig. S6. Genomic inflation plot using the combined dataset.


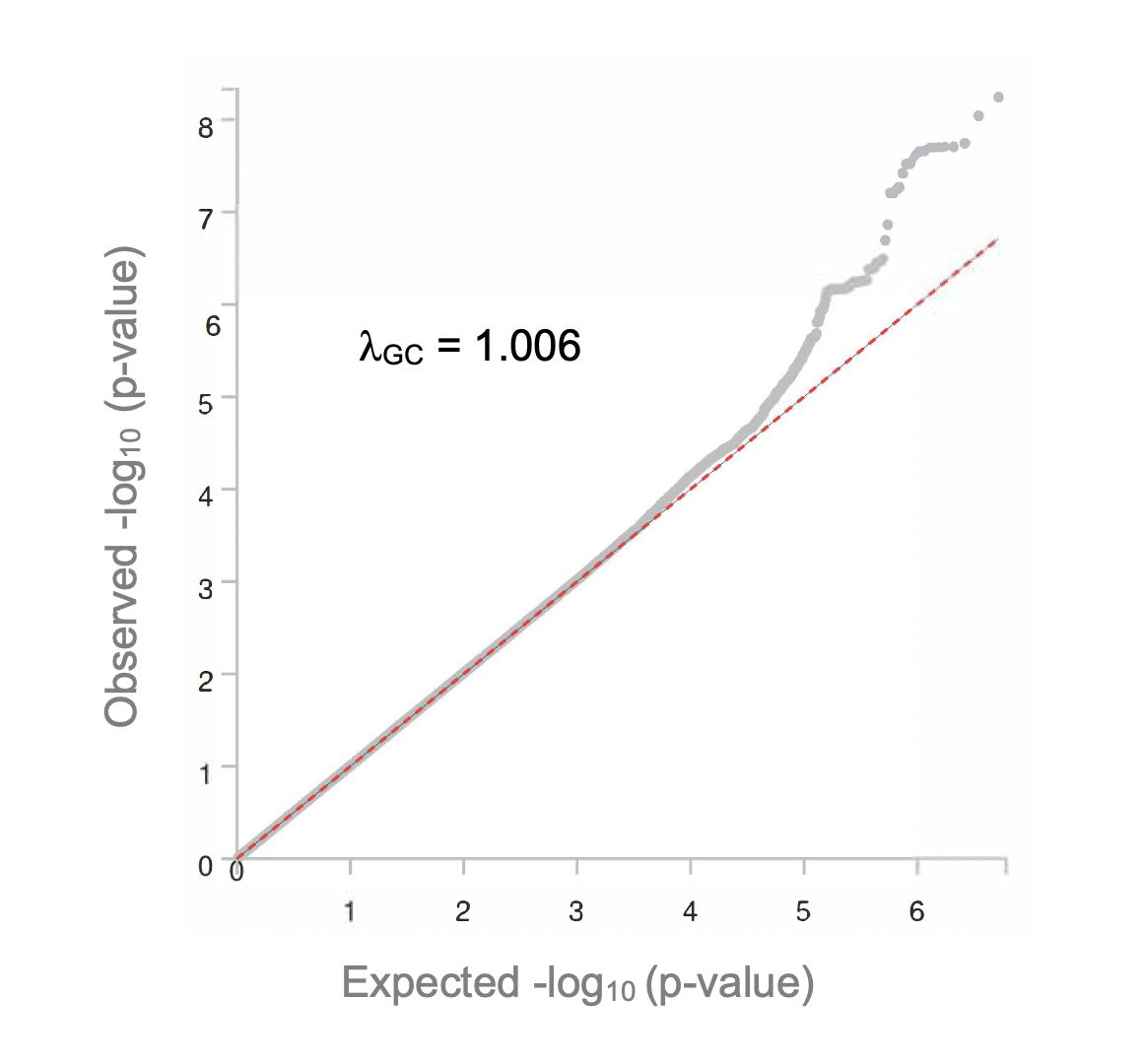


Quantile-Quantile plot showing the expected (horizontal axis) and the observed (vertical axis) quantiles of the p-values from BL GWAS with the estimated genomic inflation factor (𝜆_GC_)

## Fig. S7. Association of the top BL GWAS lead rs111457485-T stratified by country and comparison with main/original analysis.


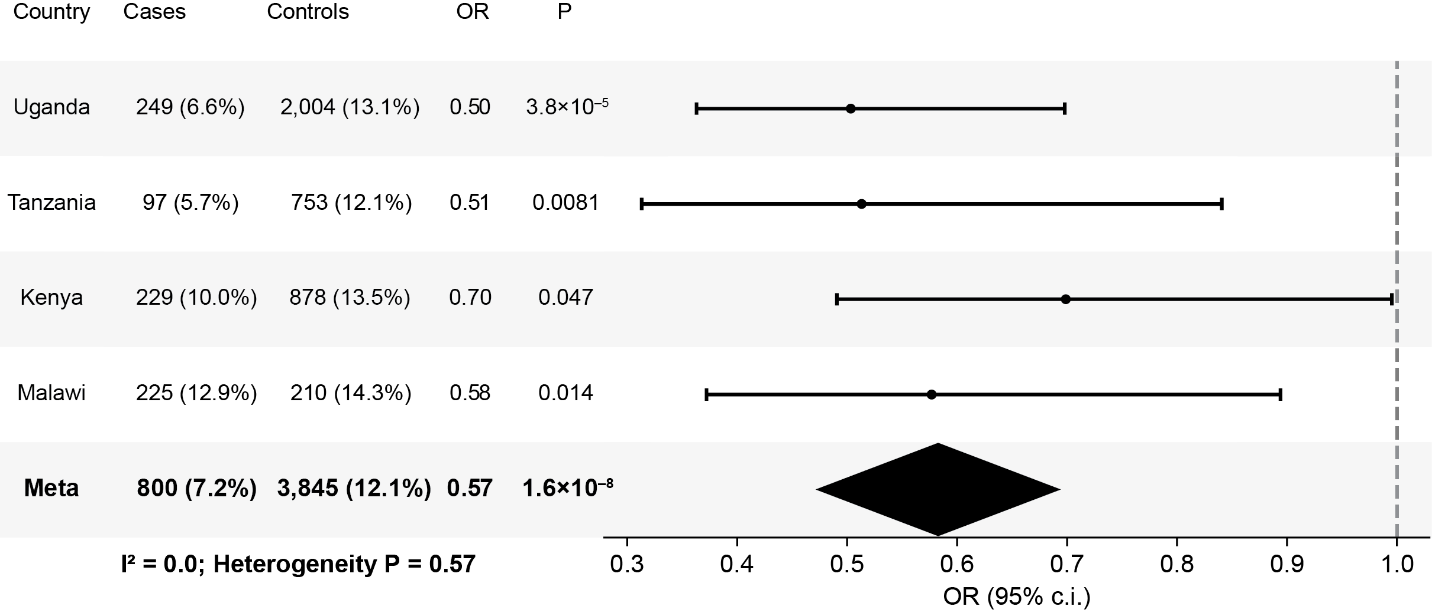


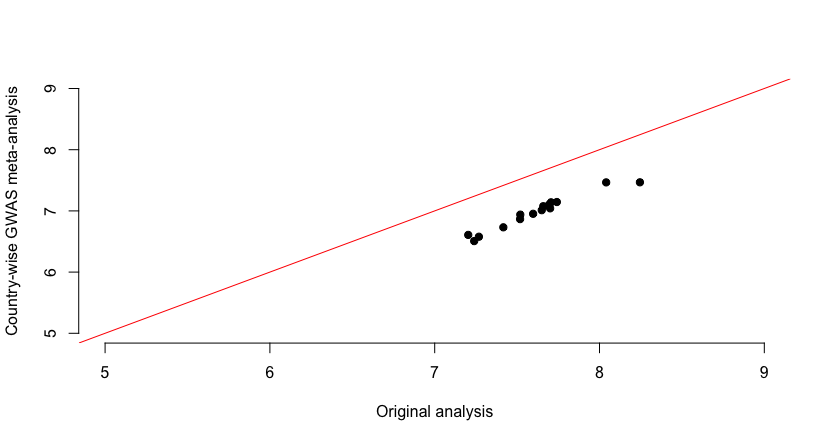


Top panel: Forest plot (odds ratio and 95% confidence interval) showing the association of the lead SNP rs111457485-T in the chr21q22.12 locus in samples across the four contributing countries as well as in the overall study. The number of cases and controls, odds ratios (ORs) and p values are displayed. The allele frequencies for rs111457485-T are shown in parentheses. Bottom panel: -log10(p-value) of the credible set SNPs in original analysis (horizontal axis) versus –log10(p-value) of the same SNPs when meta-analyzed was performed on GWAS results stratified by country, showing concordance between the results from either analysis.

## Fig. S8. The chr21q22.12 genomic region flanked by the *RUNX1* and SETD4 genes.


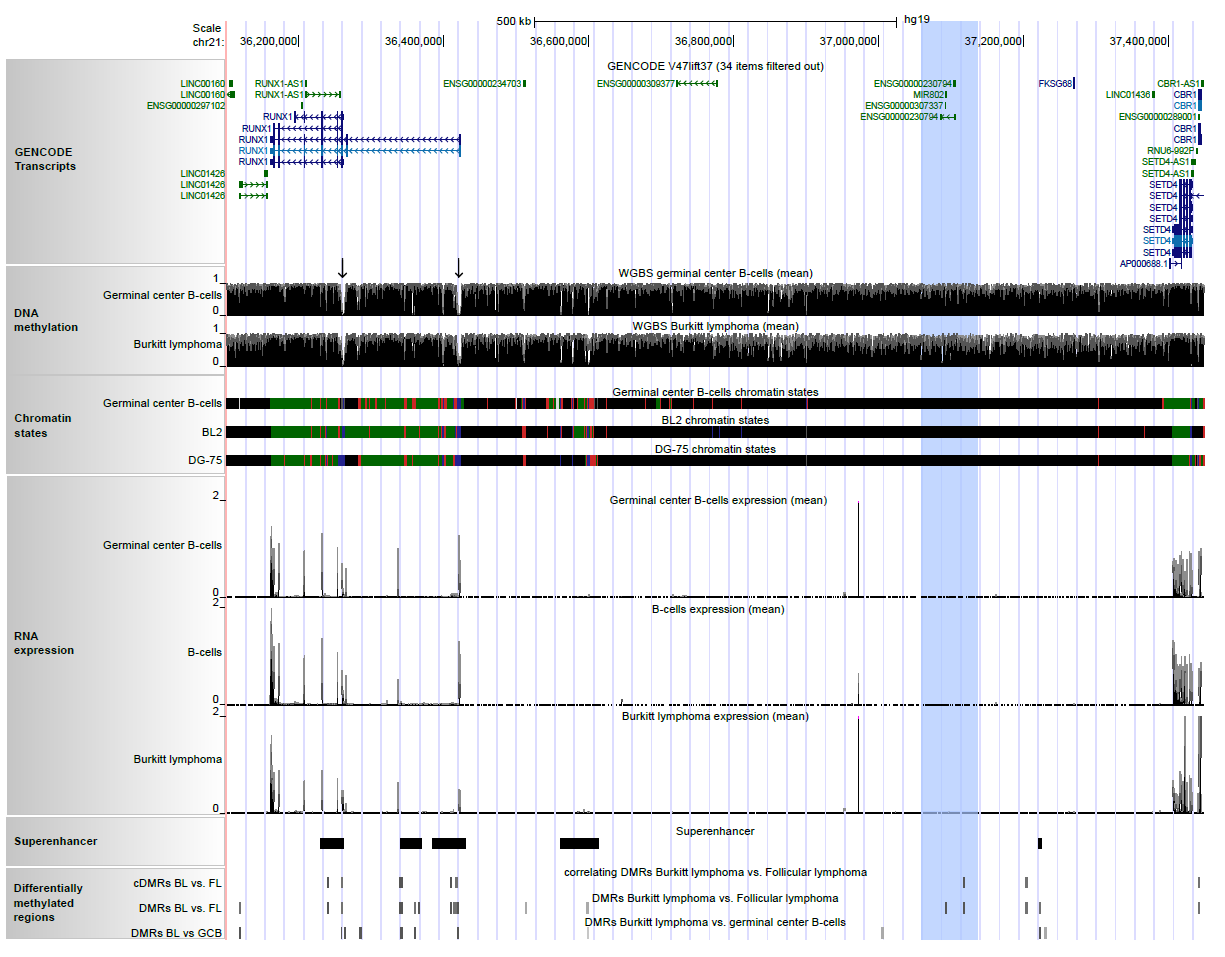
 The region with the 17 GWAS credible set SNPs associated with BL predisposition is highlighted in blue. GENCODE annotations are given below the coordinates. The WGBS tracks show the mean DNA methylation across normal germinal center B-cell (GCB) populations and primary solid BL data^36, 37^. Clear DNA methylation dips (less DNA methylation, consistent with higher gene expression) can be seen at the P1 and P2 promotors of *RUNX1* (arrows). The chromatin states are derived from the International Human Epigenome Consortium project BLUEPRINT as published ^37^. Green color indicates transcribed regions, red- enhancers, blue - promoters, black - heterochromatin. The expression tracks show mean expression measured by RNA-seq as part of the ICGC MMML-Seq project as published by Kretzmer et al.,^37^ and Lopez et al.,^38^. Coordinates of B-cell lymphomas associated superenhancers are derived from Ba et al.^36^. Differentially methylated regions (DMRs) including DMRs correlating with differential gene expression (cDMRs) between BL and follicular lymphoma (FL) or normal GCB populations are extracted from^37^. The figure is modified from UCSC Genome Browser based on GRCH37/hg19 build, chr21:36,100,000-37,450,00.

Fig. S9. Examples of significant associations between the chr21q22.12 CS-SNPs,
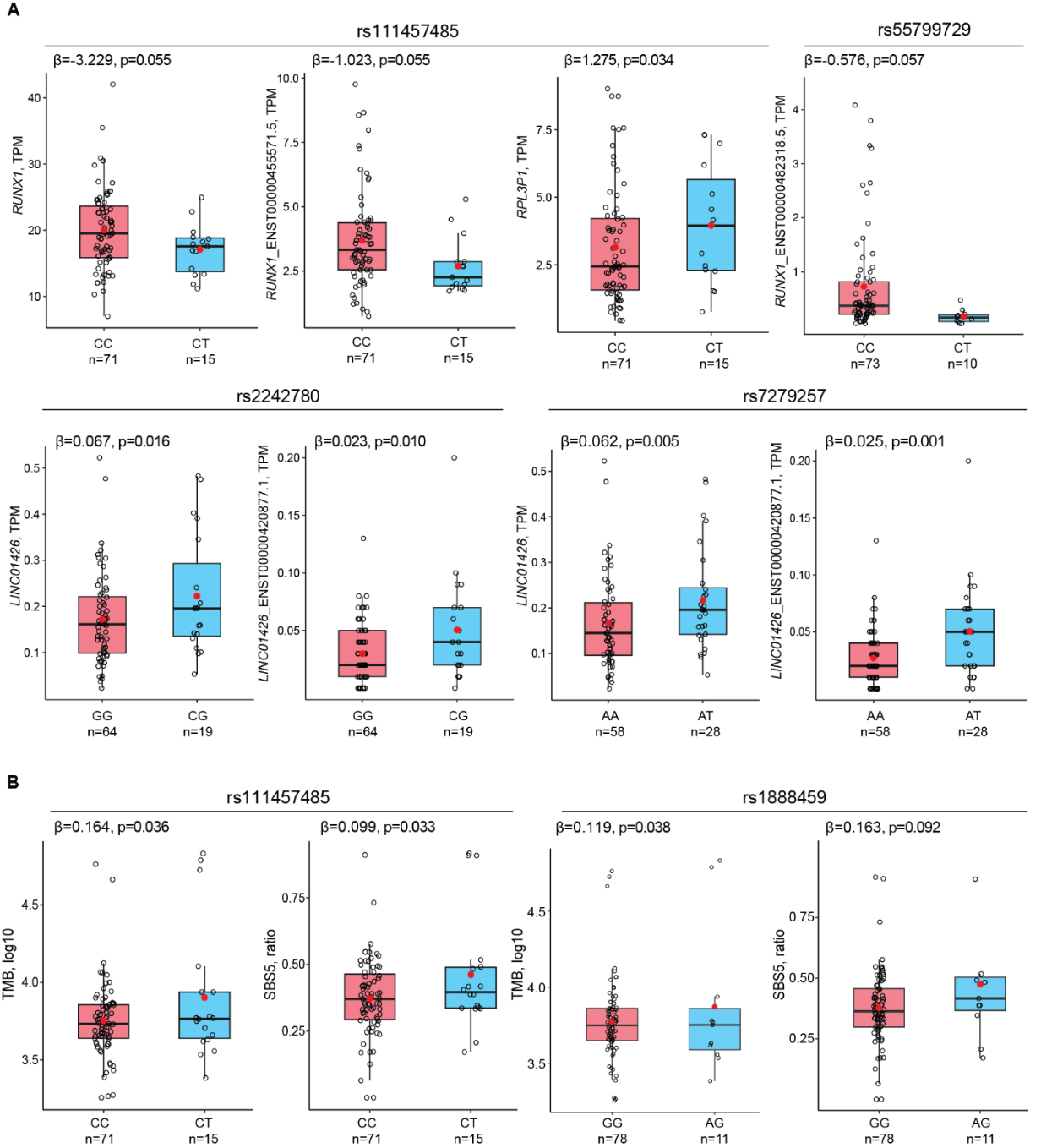
gene expression and somatic mutations.

a, Expression levels (transcripts per million, TPM); b, Tumor mutational burden (TMB, log10) and the ratio of the SBS5 mutations for select significant associations presented in **Figure 3b** for analysis in BLGSP BL tumors of patients from Uganda. The red dots indicate the group means. P values and β values are for linear regression models adjusted for sex, age, and EBV status (positive/negative). The minor alleles for each SNP are effect alleles.

## Fig. S10. Long-read RNA-sequencing in a B-cell diffuse large cell lymphoma (DLBCL) cell line OCI-LY7.


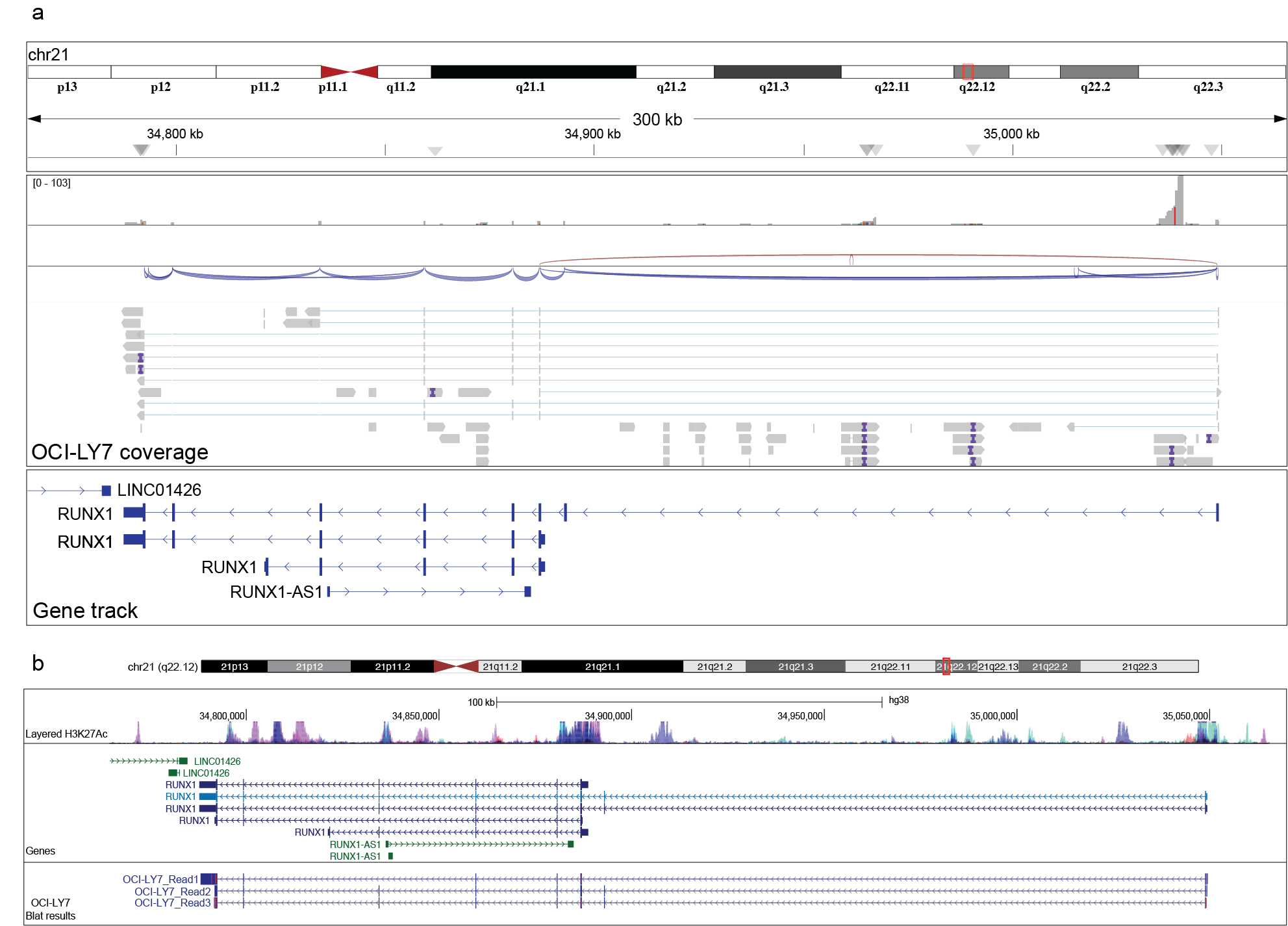


a, Results of long-read RNA sequencing of the B-cell lymphoma OCI-LY7 cell line. The IGV profile of the *RUNX1* gene region in (gene direction from right to left) showing overall read coverage and splicing junctions. b, Three individual full-length reads were extracted from long-read sequencing BAM file, and displayed via the UCSC genome browser. The reads start from the *RUNX1*-P1 promoter and span through the whole gene. All transcripts represent RUNX1c isoform from the P1 promoter, with no transcripts detected from the P2 promoter. The H3K27ac histone modification track indicates active promoter regions around the *RUNX1* gene.

## Fig. S11. Analysis of the regulatory activity of the chr21q22.12 CS-SNPs by Luciferase reporter assays.


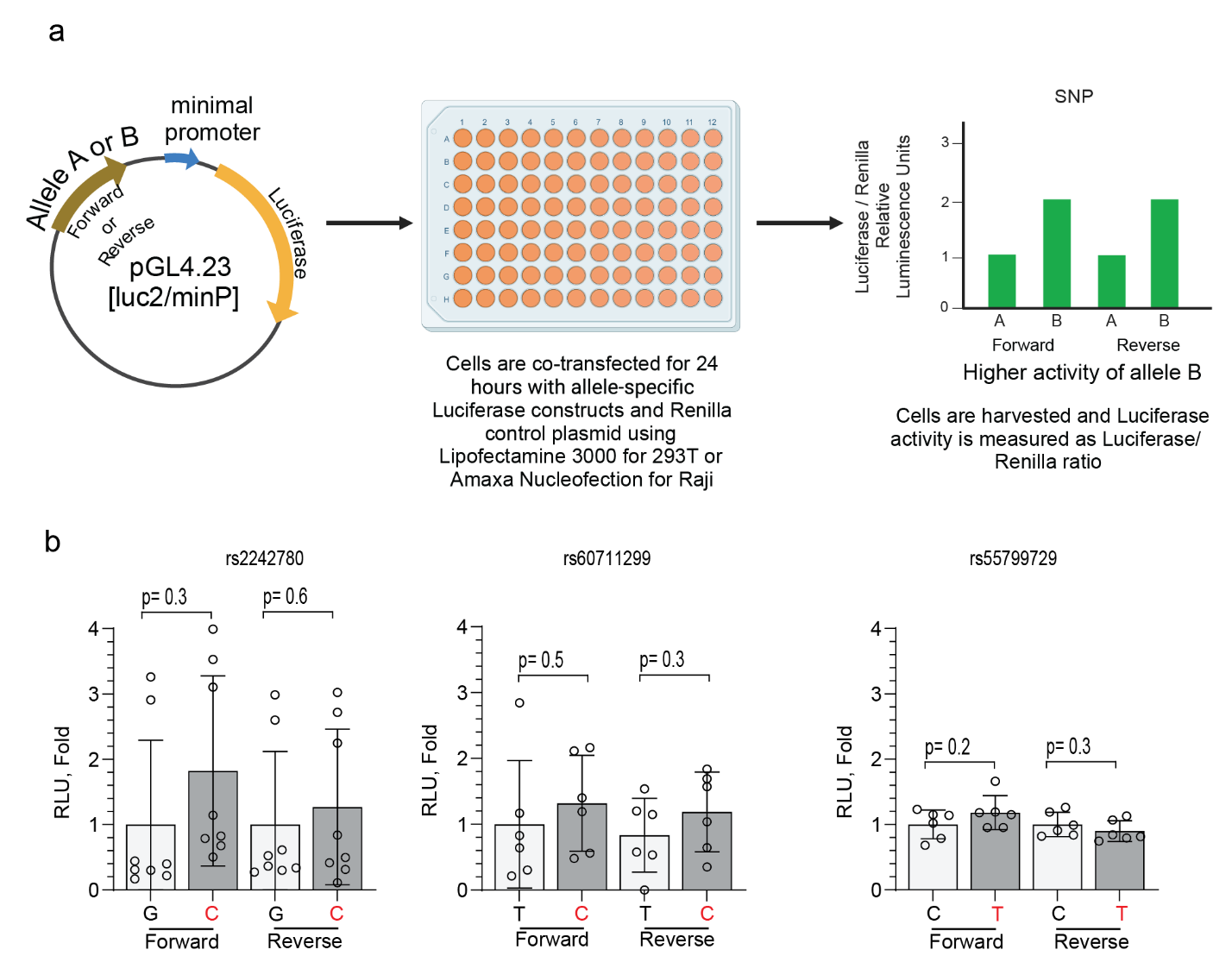


Outline of the dual-reporter Luciferase assay used for testing the regulatory activity of the 17 chr21q22.12 CS-SNPs. a, Alleles of all SNPs with ~50 bp of the surrounding sequence were cloned in forward and reverse orientations into the pGL4.23 vector with a minimal promoter. After validation, each allele-specific construct was transiently cotransfected with the Renilla control plasmid, using Lipofectamine 3000 or Amaxa Nucleofector, depending on the cell line. The cells were lysed 24 h post transfection, and the Luciferase and Renilla fluorescence levels were quantified and analyzed. Each transfection was performed in 6–12 technical replicates and repeated in 2–3 independent experiments with similar results. b, Results from one representative experiment are shown. Replication of significant results in the BL Raji cell line for Luciferase reporter assays detected in the HEK293 cells (**Figure 3c**). The effect alleles of each SNP are shown in red.

## Fig. S12. The evaluation of DNA‒protein interactions of regulatory chr21q22.12 CS-SNPs by electrophoretic mobility shift assays (EMSA).


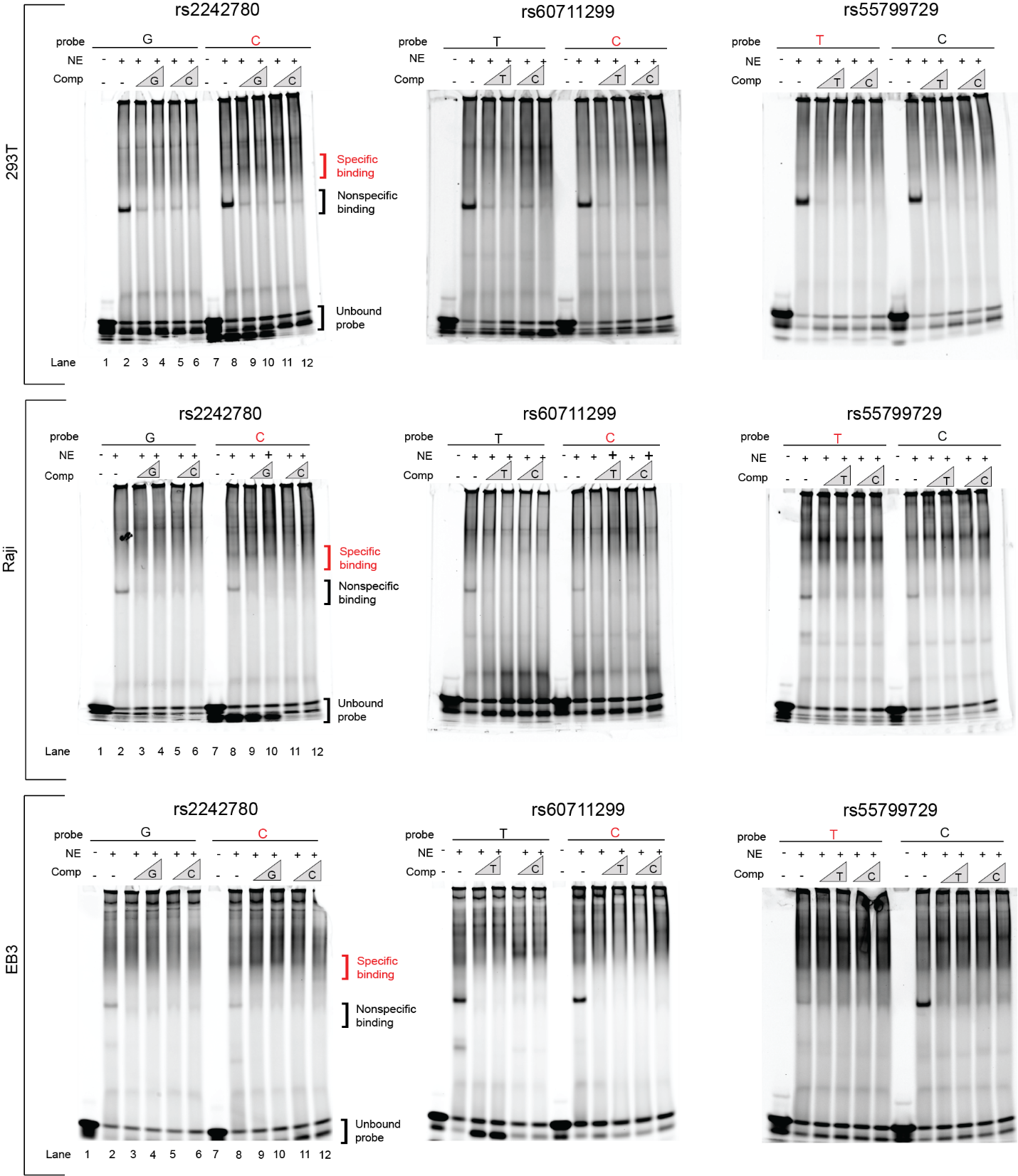


The results of electrophoretic mobility shift assays (EMSA) using nuclear cell extracts from HEK293T, Raji, and EB-3 cell lines for the three SNPs with regulatory activity identified by the dual-luciferase reporter assays. The protective alleles of each SNP are labeled using red font letters. On each plot, lanes 1 and 7 include only labeled DNA probe with indicated allele, lanes 2 and 8 include labeled DNA probe with nuclear extract (NE) from the corresponding cell line; lanes 3-6 and 9-12 include 100x excess of unlabeled self-probe (specific competitor) or non-self-probe (non-specific competitor) added to the reaction. Competition with unlabeled self-oligo should reduce the intensity of the band shifted due to protein binding. The red brackets are used to mark specific binding for the rs2242780-C allele. The bands detected in lanes for both alleles are due to “non-specific binding” as marked by black brackets for the rs2242780-C allele. The excess of labeled probes not used in the reaction are labeled as “unbound probe”. The results shown are from one representative experiment of at least 3 independent experiments.

## Fig. S13. The genomic landscape of the chr21q22.12 BL GWAS locus.


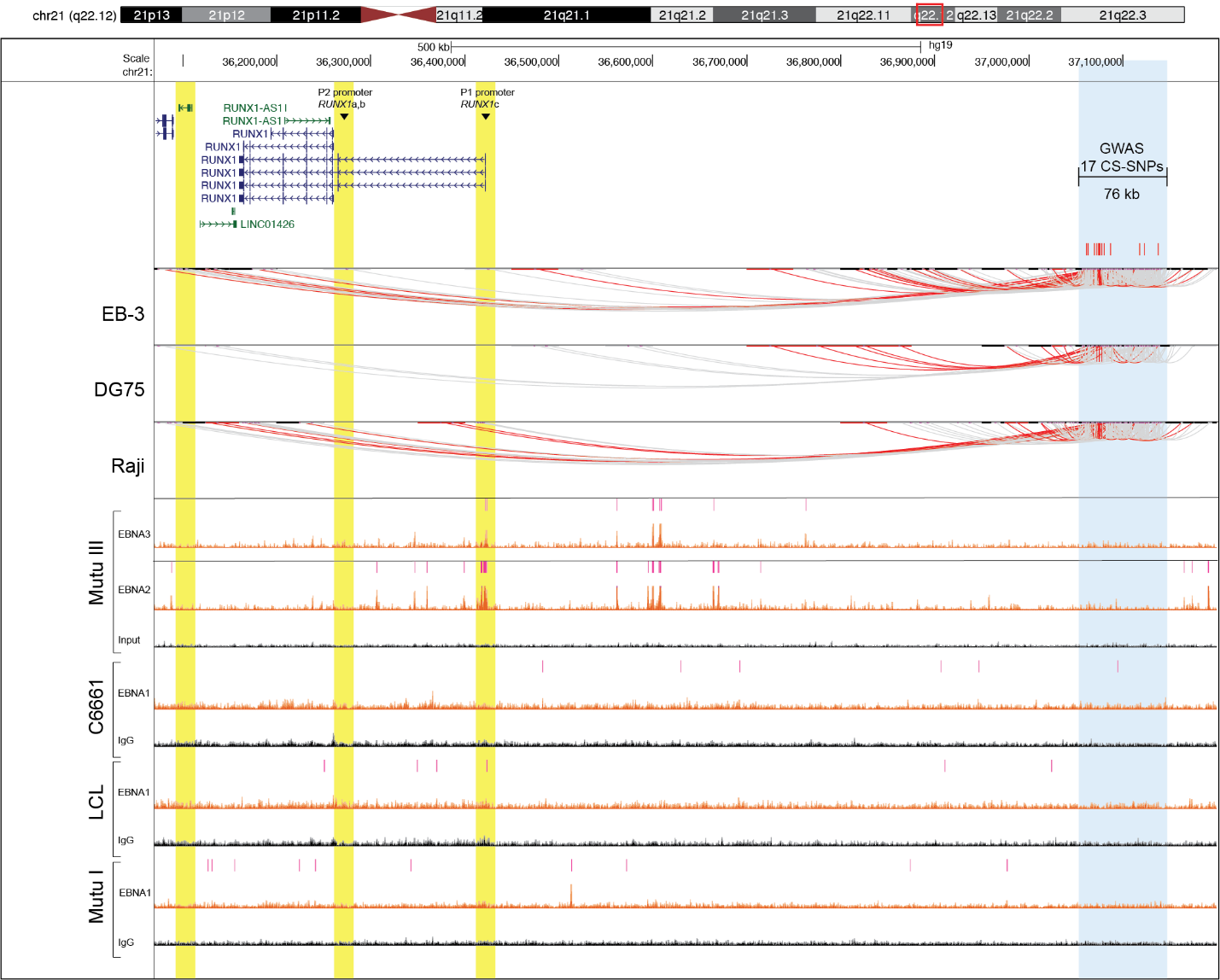


Map of chromatin interactions in BL cell lines: EB-3 (heterozygous), DG75 and Raji (negative) for minor effect alleles of all 17 chr21q22.12 CS-SNPs. The *RUNX1* promoters P1 (producing the *RUNX1c* transcript) and P2 (producing the *RUNX1a* and *RUNX1b* transcripts) and the promoter of *LINC01426* are highlighted in yellow. Long-range chromatin interactions between the 76 Kb GWAS CS-SNP region (highlighted in blue) and surrounding areas were determined by Pore-seq and visualized by loops; red loops originate within the ~2 Kb genomic region that includes SNPs rs111457485 and rs2242780, and gray loops indicate any other interactions. The map of ChIP-seq peaks for EBNA1, ENBA2 and EBNA3 in the BL cell lines Mutu III, a lymphoblastoid cell line (LCL), undifferentiated nasopharyngeal carcinoma cell line C6661, and Mutu I BL cell line shows binding sites around the *RUNX1 P1* promoter but not within the GWAS CS-SNP area.

## Fig. S14. Flow chart showing retrospective follow-up of 249 Ugandan BL patients to determine vital status and chemotherapy information.


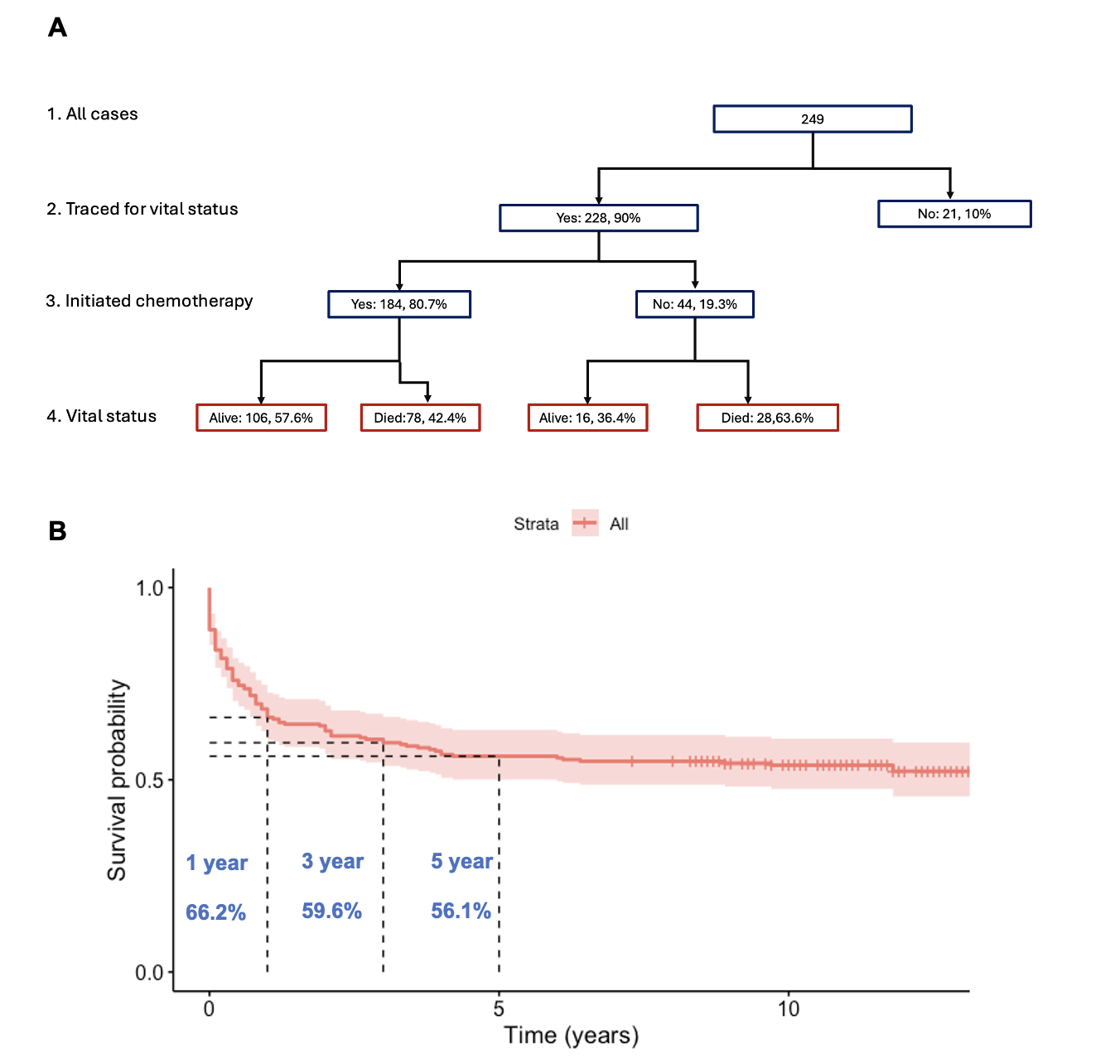


a, Flow chart showing the Ugandan BL cases that were retrospectively traced for vital status, with medical charts abstracted for chemotherapy information; b, Kaplan‒Meier curve for overall survival in 228 cases with available vital status data showing the 1-, 3- and 5-year overall survival (OS) probabilities for BL patients.

## Fig. S15. Overall survival (OS) of 228 Ugandan patients after BL diagnosis according to select characteristics.


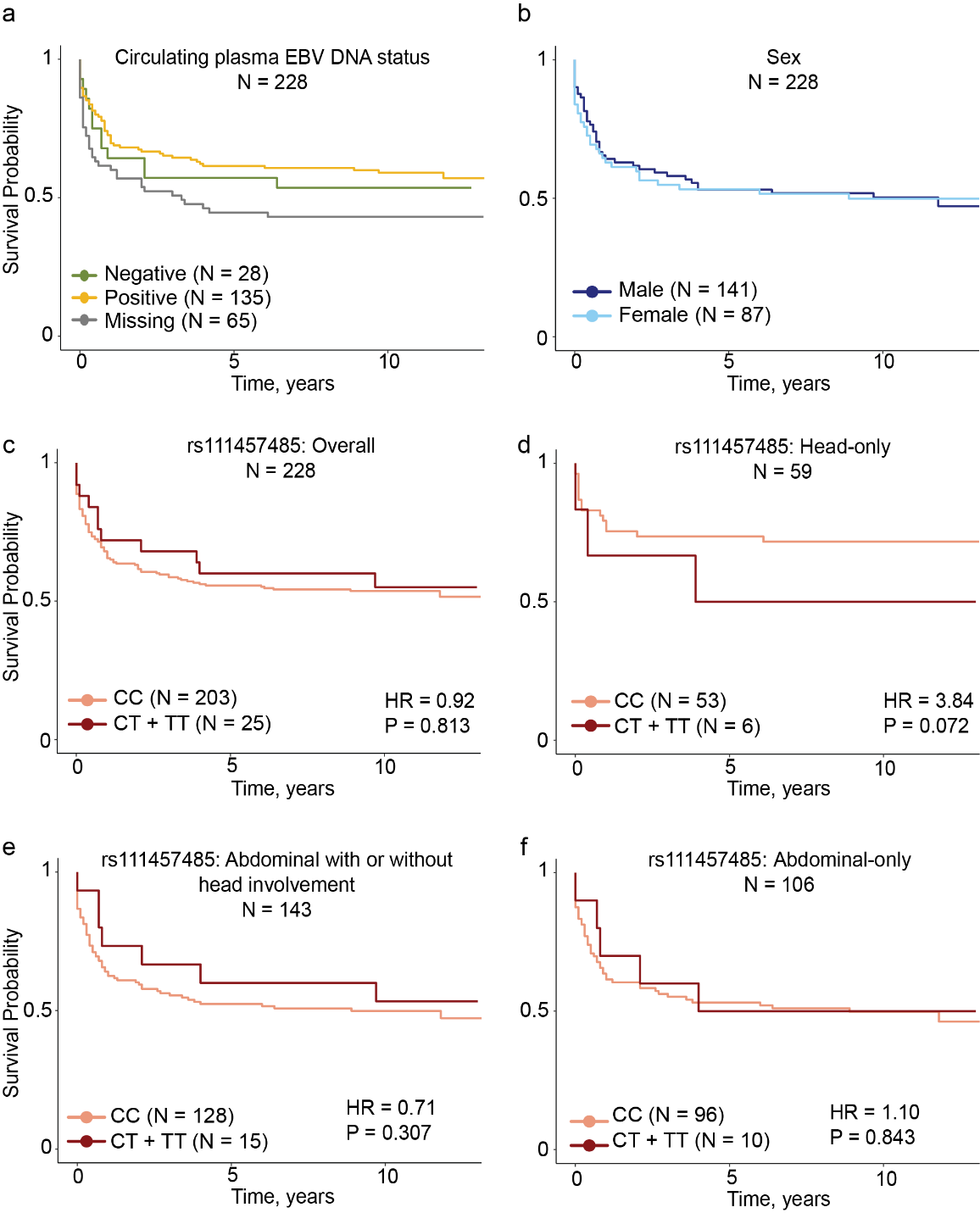


Hazard ratios (HRs) and log-rank test *P* values for OS by: a, circulating plasma EBV DNA status (reference: EBV-positive); b**,** sex (reference: female); c, rs111457485 (reference: CC genotype, BL risk in GWAS) for all cases (n=228); d, for head-only BL cases (n = 59); e, for abdominal BL with or without involvement of the head (n = 143); f, for abdominal-only BL (n = 106), (**Supplementary Table S6**).

## Fig. S16. A suggestive 3q26.1 GWAS locus tagged by the SNP rs9847876.


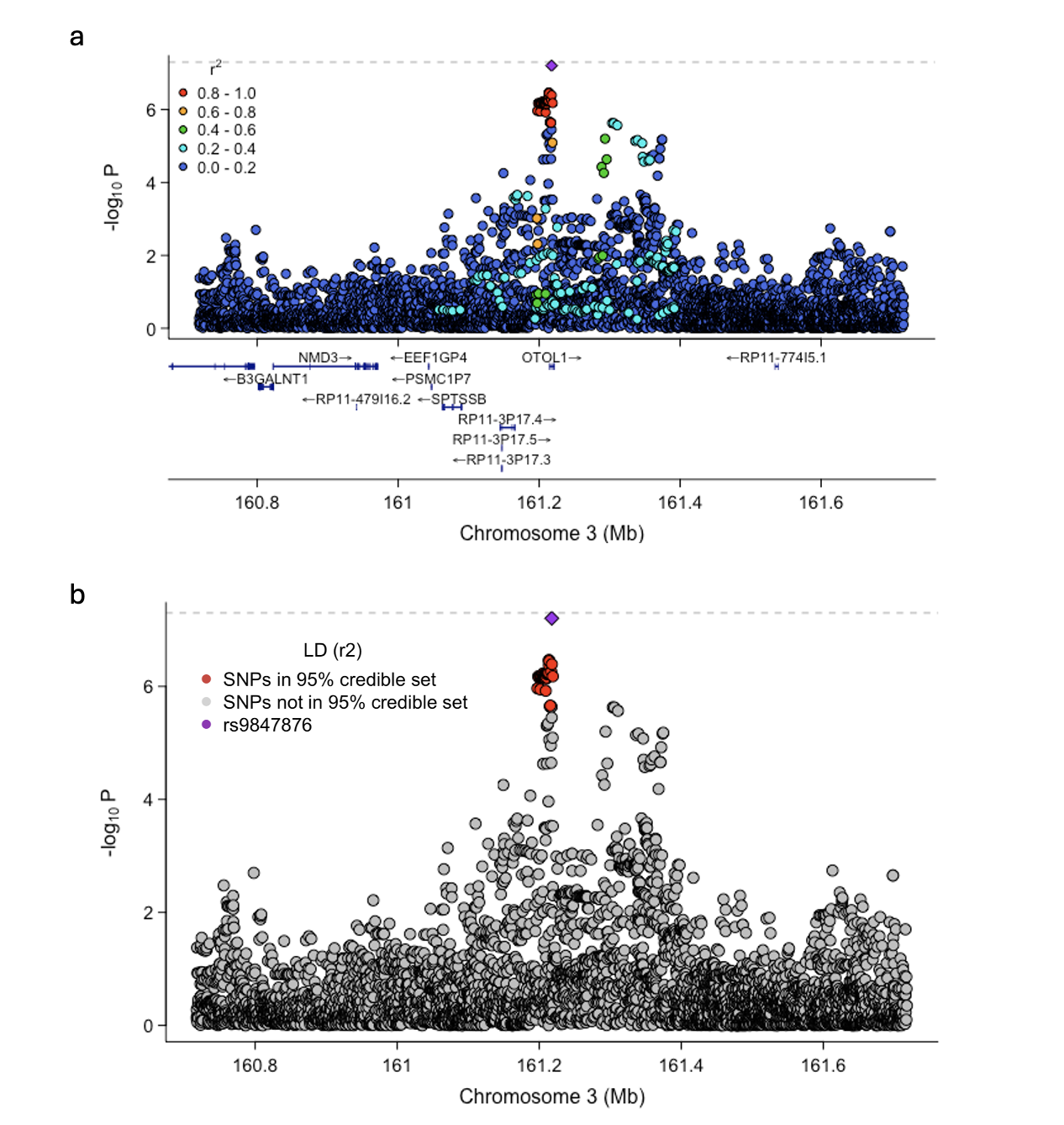


a, Regional plot for the suggestive locus at 3q26.1 with the index SNP rs9847876 (OR=0.71, *P*=6.2×10^-8^). Markers are colored based on LD (r^2^) with rs9847876 in the GWAS set (N = 4,625). b, 95% credible set for the suggestive signal at chr 3q26.26 comprising 48 SNPs. The dashed lines indicate the genome-wide significance threshold (-log10 *P*-value=5x10^-8^).

## Fig. S17. Additional BL-GWAS signals within the 19p13.2 locus identified by multi-marker tests.


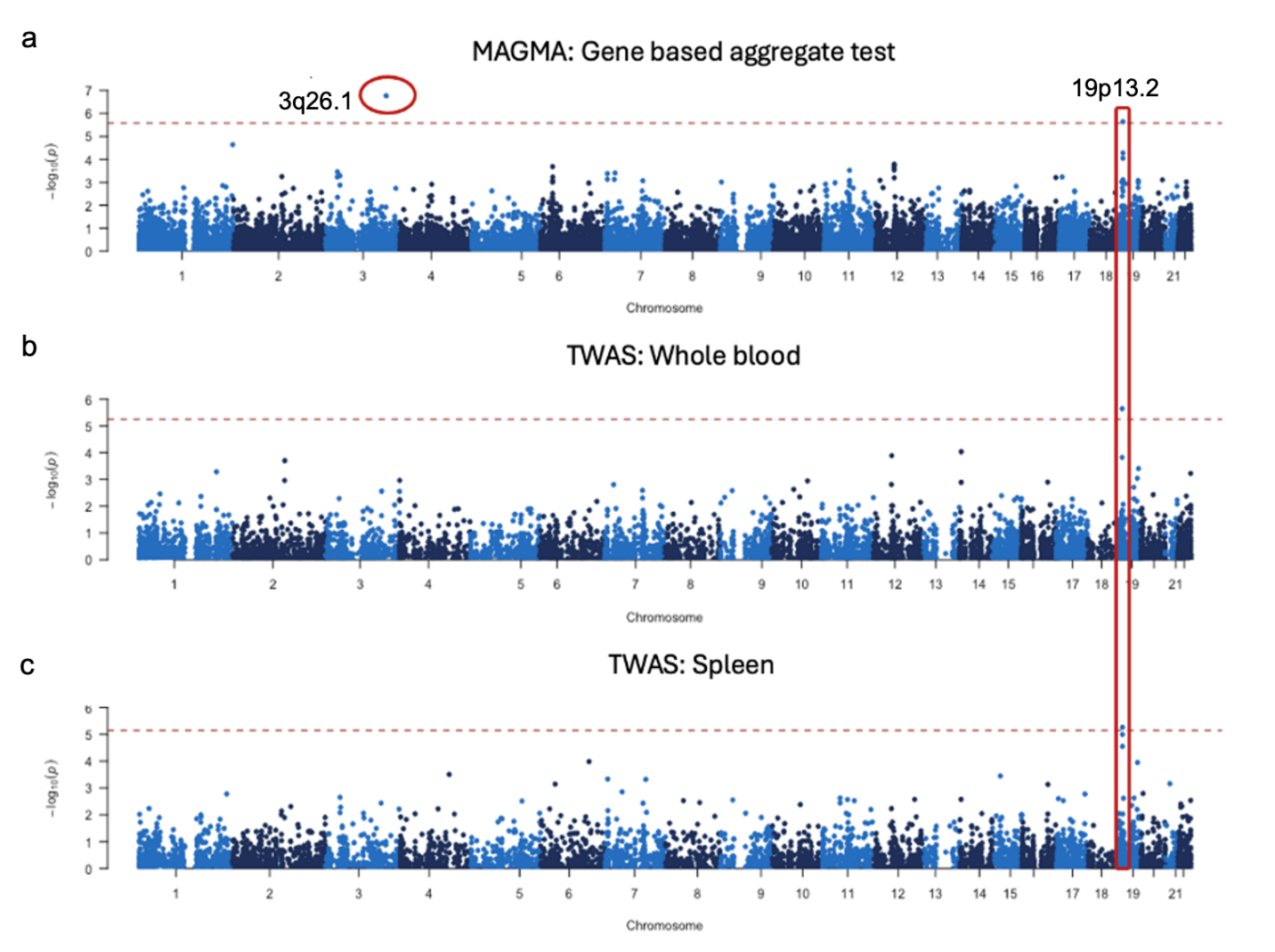


a, Gene-based multi-marker association tests from MAGMA. TWAS using gene-expression models from GTEx: b, whole blood, n=670; c, spleen, n=227. The dotted lines indicate the Bonferroni corrected significance thresholds for respective analyses: -log10 *P*-value= 2.5×10^-6^ (MAGMA, 20,000 protein-coding genes), -log10 *P*-value= 5.7×10^-6^ (TWAS in whole blood, 8799 genes profiled) and -log10 *P*-value= 7.1×10^-6^ (TWAS in spleen, 7097 genes profiled). The significant loci are marked.

## Fig. S18. Comparative analysis of the rs2242780 genomic region.


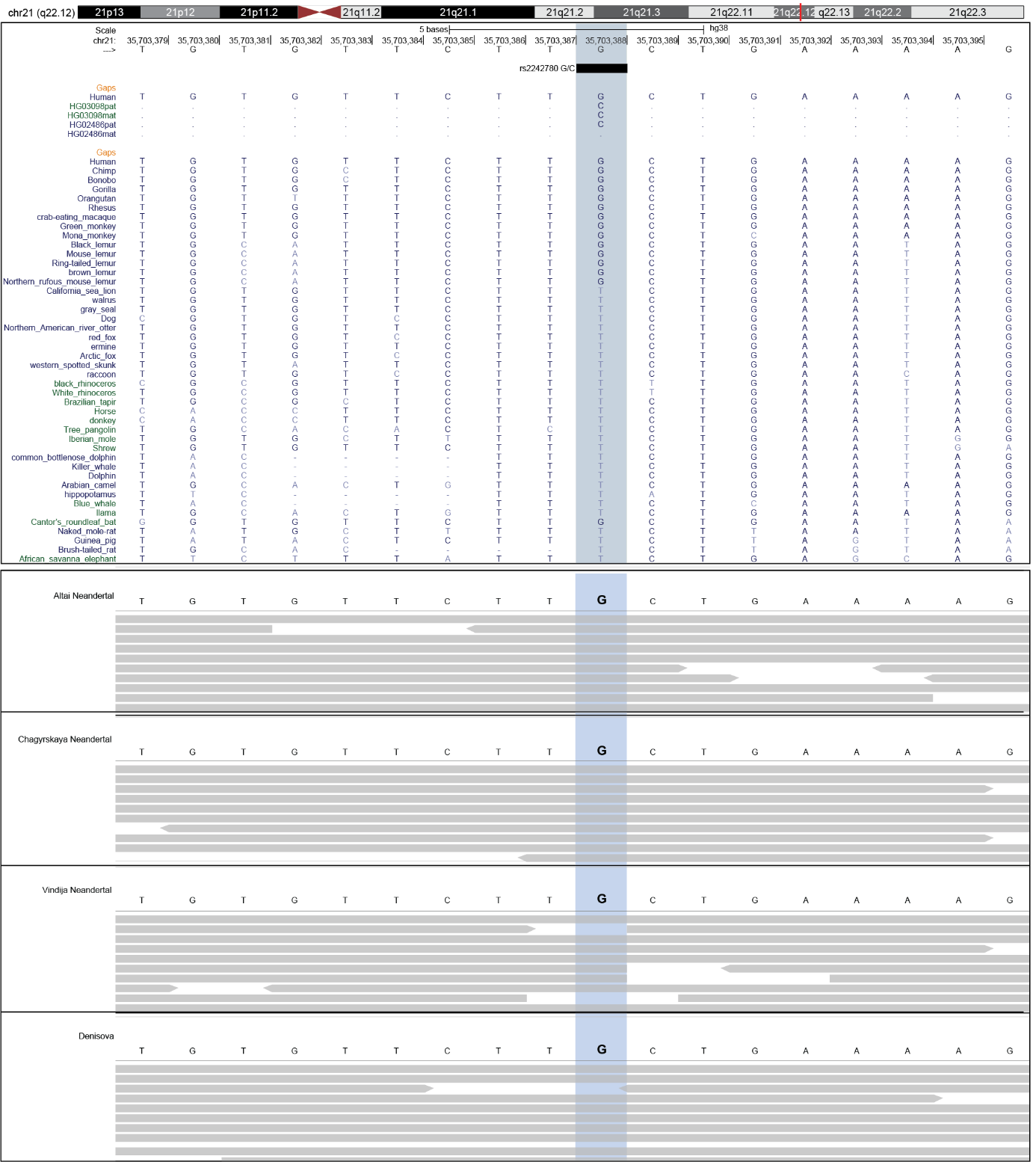


The human reference genome includes the ancestral rs2242780-G allele, whereas the HPRC individuals HG03098 (Mende from Sierra Leone) and HG02486 (African Caribbean in Barbados) carry the derived rs2242780-C allele. The C allele is not found in any species shown or archaic humans (Neandertal or Denisova).

## Fig. S19. A proposed model to explain the associations for rs111457485-T and rs2242780-C with BL risk and survival.


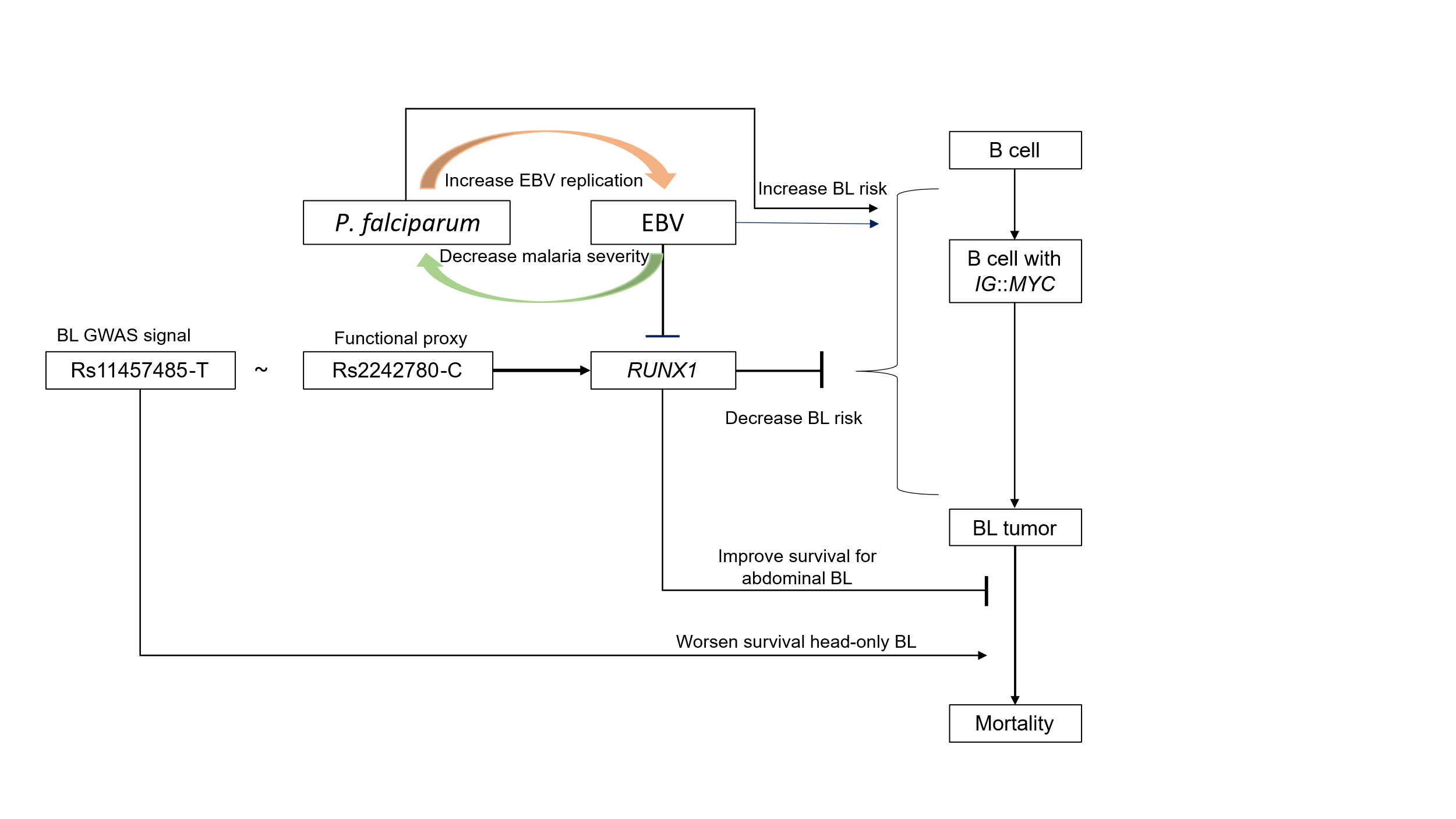


We hypothesize that rs2242780-C is the functional proxy for the BL-protective association with rs11457485-T allele, and the protective association with BL mortality in patients with abdominal tumors. The rs11457485-T was associated with worse OS for head-only BL, suggesting that this allele might have other biological effects. The graphic shows the interplay with known risk factors -*P. falciparum* and EBV, which likely influence the risk of B cells acquiring *IG***::***MYC* translocations and cooperating mutations associated with BL^39^. The cartoon shows a synergistic relationship between EBV and *P. falciparum* posited by Watier *et al.*^40^, where *P. falciparum* upregulates EBV lytic replication, which increases the burden of EBV in latently infected B cells and the amount of virus shed in saliva for transmission to new hosts. Coincidently, EBV may be protective against severe malaria through expression of viral homologs with anti-inflammatory activity, e.g., BCRF-1, an IL-10 -related peptide, which may not only increase the survival of children predisposed to BL but could also contribute to the inverse association between *P. falciparum* parasitemia and malaria observed in ours and other studies. If so, the direct effects of *P. falciparum* shown in mouse models^41^ may be attributed to frequent but low-grade asymptomatic infections.

## Fig. S20. Evaluation of batch effects in Uganda genotype results.


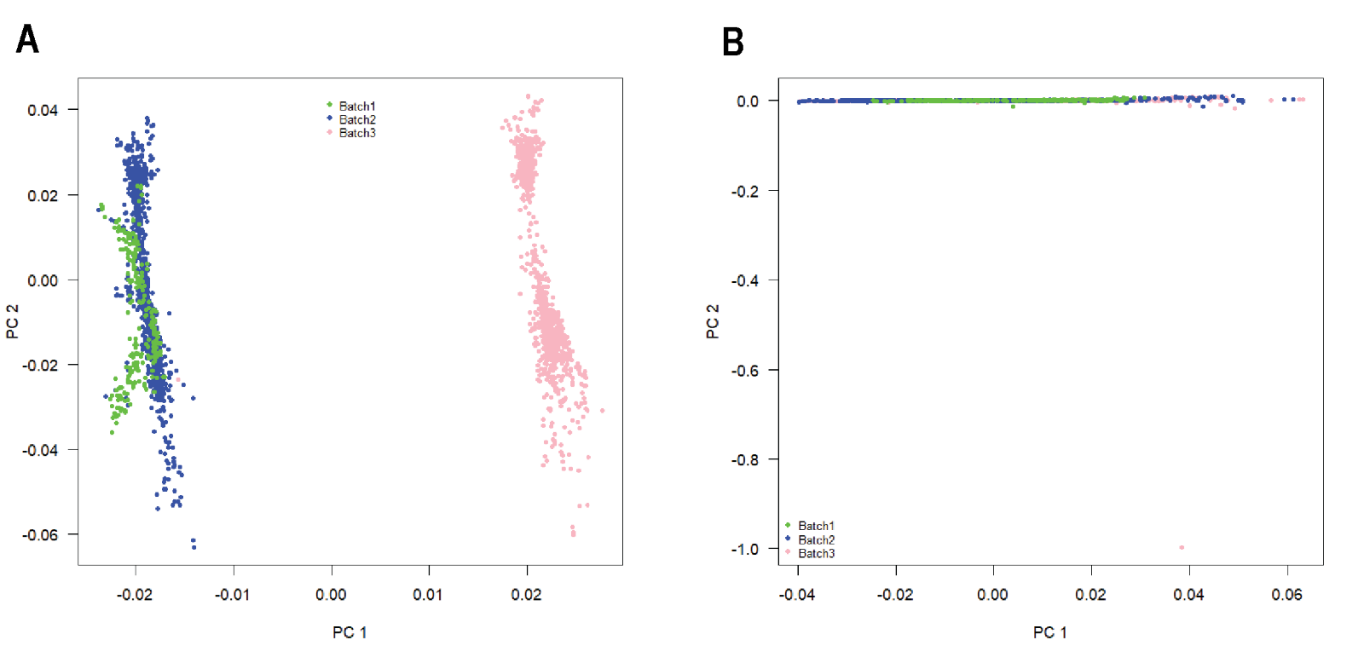


a, Principal component analysis (PCA) using 4,143,558 variants after minimal variant quality control (--geno 0.05) for samples in Uganda tested in three batches; b, PC1 versus PC2 plots after removing 27,039 batch effect SNPs showing that remaining SNPs cluster together along the PC1 gradient. One outlier individual is noted, perhaps due to different reasons, e.g., ancestry or missing data, but does not cause batch clustering of SNPs, and is not observed in in PCA with standard filtering, including MAF and HWE in Fig. S2)

## Fig. S21. Principal Component Analysis to assess substructure of BL cases analyzed by select characteristics.


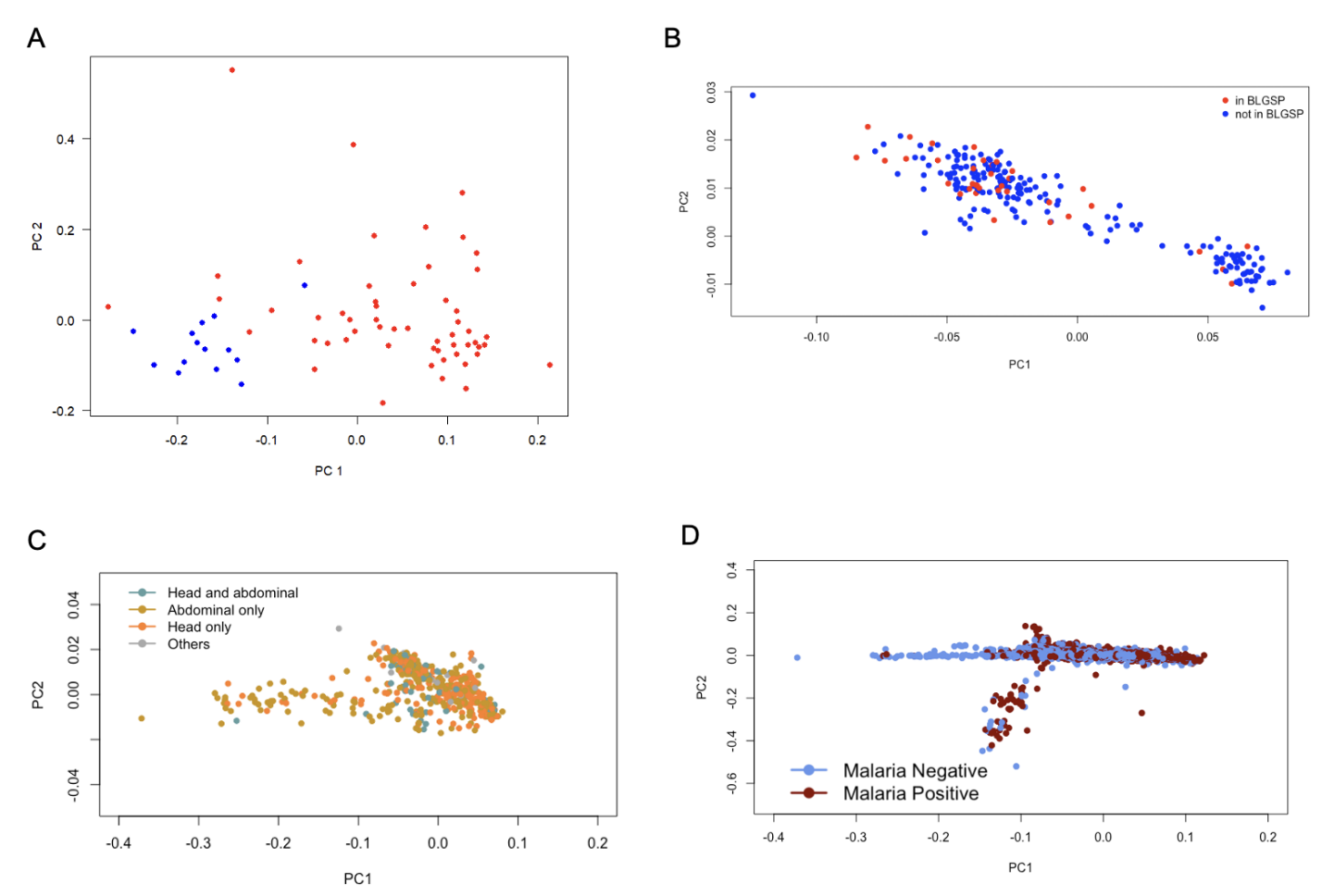


a, PCA using 727,834 uncorrelated SNPs to assess the substructure between EMBLEM patients enrolled in (red dots) versus those not enrolled (blue dots) in the BLGSP; b, PCA using 393,851 uncorrelated SNPs from whole exome sequencing data to assess the substructure between BLGSP patients from EMBLEM (blue dots) versus BLGSP cases from another site in Uganda that were not part of EMBLEM (red dots). c, PCA values restricted to 512 cases colored by anatomical sites of BL d, PCA values restricted to and colored by samples with *P. falciparum* infection status. Overlapping distributions suggest similarities in ancestries and geographic areas for the sets of patients being compared.

## Fig. S22. Distribution of anatomic sites in 512 BL patients from the EMBLEM study with data, by enrollment year.


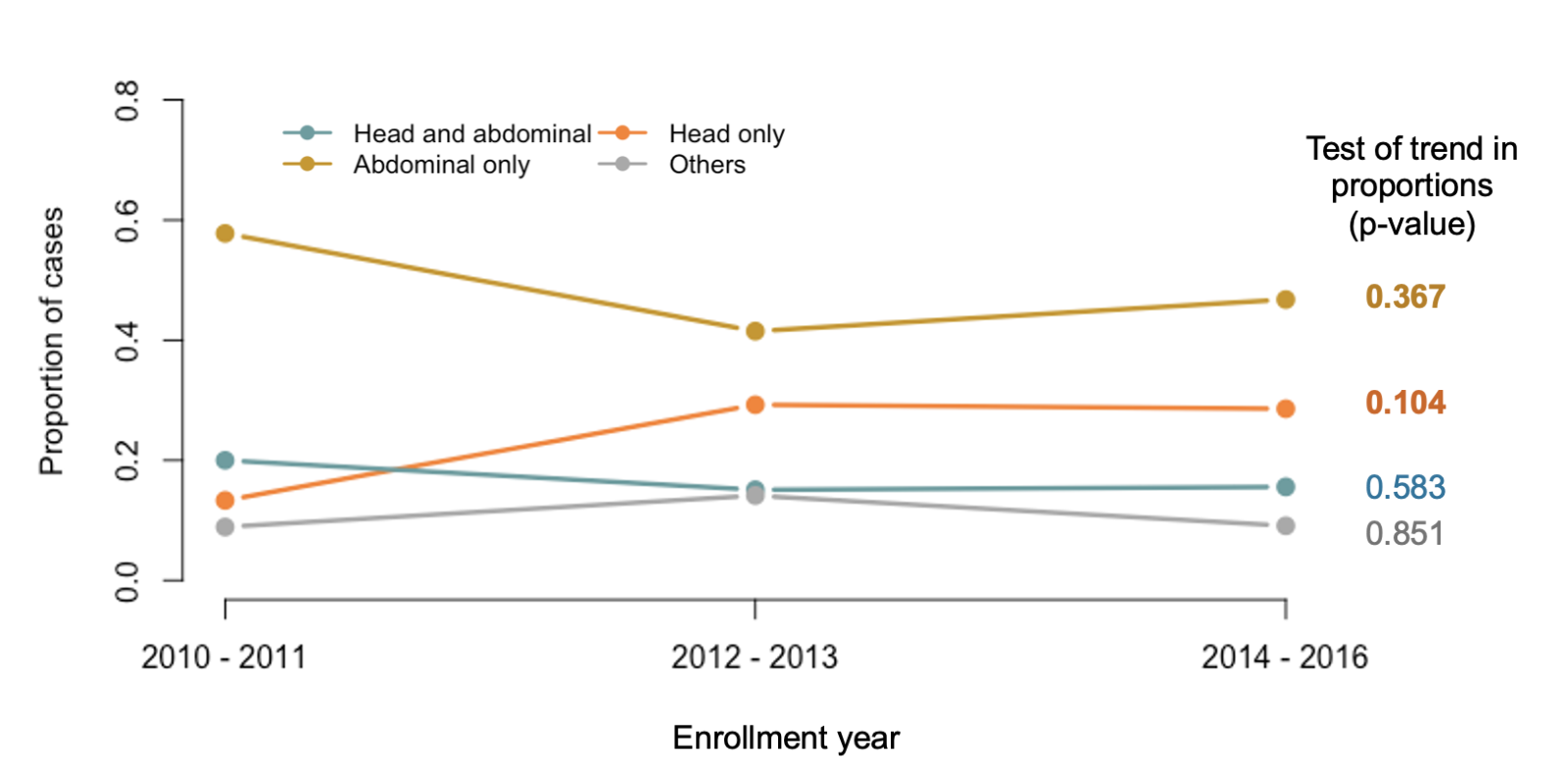


The proportion of cases (%) with tumor anatomic sites classified into four categories and analyzed by enrollment year. *P*-values are for the chi-squared test comparing to the 2010–2012 interval. Anatomic site information was collected in EMBLEM using an Anatomic Site Form: https://emblem.cancer.gov/resources/forms_case/Tumor_Anatomic_Site-HIPE2.pdf. Anatomic site information was not available for patients in Malawi.

# Section -3 References

1. Peprah S, Ogwang MD, Kerchan P, Reynolds SJ, Tenge CN, Were PA*, et al.* Risk factors for Burkitt lymphoma in East African children and minors: A case-control study in malaria-endemic regions in Uganda, Tanzania and Kenya. *Int J Cancer* 2020 Feb 15; **146**(4)**:** 953-969.

2. Mutalima N, Molyneux E, Jaffe H, Kamiza S, Borgstein E, Mkandawire N*, et al.* Associations between Burkitt lymphoma among children in Malawi and infection with HIV, EBV and malaria: results from a case-control study. *PLoS One* 2008; **3**(6)**:** e2505.

3. Hong HG, Gouveia MH, Ogwang MD, Kerchan P, Reynolds SJ, Tenge CN*, et al.* Sickle cell allele HBB-rs334(T) is associated with decreased risk of childhood Burkitt lymphoma in East Africa. *Am J Hematol* 2024 Jan; **99**(1)**:** 113-123.

4. Grande BM, Gerhard DS, Jiang AX, Griner NB, Abramson JS, Alexander TB*, et al.* Genome-wide discovery of somatic coding and noncoding mutations in pediatric endemic and sporadic Burkitt lymphoma. *Blood* 2019 Mar 21; **133**(12)**:** 1313-1324.

5. Ziegler JL. Burkitt's Lymphoma. *New England Journal of Medicine* 1981; **305**(13)**:** 735-745.

6. Nkrumah FK, Perkins IV. Burkitt's lymphoma. A clinical study of 110 patients. *Cancer* 1976; **37**(2)**:** 671-676.

7. Olweny CL, Katongole-Mbidde E, Otim D, Lwanga SK, Magrath IT, Ziegler JL. Long-term experience with Burkitt's lymphoma in Uganda. *Int J Cancer* 1980 Sep 15; **26**(3)**:** 261-266.

8. Gupta S, Aitken JF, Bartels U, Brierley J, Dolendo M, Friedrich P*, et al.* Paediatric cancer stage in population-based cancer registries: the Toronto consensus principles and guidelines. *Lancet Oncol* 2016 Apr; **17**(4)**:** e163-e172.

9. Mbulaiteye SM, Katabira ET, Wabinga H, Parkin DM, Virgo P, Ochai R*, et al.* Spectrum of cancers among HIV-infected persons in Africa: the Uganda AIDS-Cancer Registry Match Study. *Int J Cancer* 2006 Feb 15; **118**(4)**:** 985-990.

10. Liu Z, Luo Y, Kirimunda S, Verboom M, Onabajo OO, Gouveia MH*, et al.* Human leukocyte antigen-DQA1*04:01 and rs2040406 variants are associated with elevated risk of childhood Burkitt lymphoma. *Commun Biol* 2024 Jan 5; **7**(1)**:** 41.

11. Arisue N, Chagaluka G, Palacpac NMQ, Johnston WT, Mutalima N, Peprah S*, et al.* Assessment of Mixed Plasmodium falciparum sera5 Infection in Endemic Burkitt Lymphoma: A Case-Control Study in Malawi. *Cancers (Basel)* 2021 Apr 2; **13**(7).

12. Gouveia MH, Bergen AW, Borda V, Nunes K, Leal TP, Ogwang MD*, et al.* Genetic signatures of gene flow and malaria-driven natural selection in sub-Saharan populations of the "endemic Burkitt Lymphoma belt". *PLoS Genet* 2019 Mar; **15**(3)**:** e1008027.

13. Zhou W, Fischer A, Ogwang MD, Luo W, Kerchan P, Reynolds SJ*, et al.* Mosaic chromosomal alterations in peripheral blood leukocytes of children in sub-Saharan Africa. *Nat Commun* 2023 Dec 6; **14**(1)**:** 8081.

14. Purcell S, Neale B, Todd-Brown K, Thomas L, Ferreira MA, Bender D*, et al.* PLINK: a tool set for whole-genome association and population-based linkage analyses. *Am J Hum Genet* 2007 Sep; **81**(3)**:** 559-575.

15. Loh PR, Danecek P, Palamara PF, Fuchsberger C, Y AR, H KF*, et al.* Reference-based phasing using the Haplotype Reference Consortium panel. *Nat Genet* 2016 Nov; **48**(11)**:** 1443-1448.

16. Durbin R. Efficient haplotype matching and storage using the positional Burrows-Wheeler transform (PBWT). *Bioinformatics* 2014 May 1; **30**(9)**:** 1266-1272.

17. Leal TP, Furlan VC, Gouveia MH, Saraiva Duarte JM, Fonseca PA, Tou R*, et al.* NAToRA, a relatedness-pruning method to minimize the loss of dataset size in genetic and omics analyses. *Comput Struct Biotechnol J* 2022; **20:** 1821-1828.

18. Zhou W, Bi W, Zhao Z, Dey KK, Jagadeesh KA, Karczewski KJ*, et al.* SAIGE-GENE+ improves the efficiency and accuracy of set-based rare variant association tests. *Nat Genet* 2022 Oct; **54**(10)**:** 1466-1469.

19. Puhr R, Heinze G, Nold M, Lusa L, Geroldinger A. Firth's logistic regression with rare events: accurate effect estimates and predictions? *Stat Med* 2017 Jun 30; **36**(14)**:** 2302-2317.

20. Wang G, Sarkar A, Carbonetto P, Stephens M. A Simple New Approach to Variable Selection in Regression, with Application to Genetic Fine Mapping. *Journal of the Royal Statistical Society Series B: Statistical Methodology* 2020; **82**(5)**:** 1273-1300.

21. Liao W-W, Asri M, Ebler J, Doerr D, Haukness M, Hickey G*, et al.* A draft human pangenome reference. *Nature* 2023 2023/05/01; **617**(7960)**:** 312-324.

22. Florez-Vargas O, Ho M, Hogshead MH, Papenberg BW, Lee CH, Forsythe K*, et al.* Genetic regulation of TERT splicing affects cancer risk by altering cellular longevity and replicative potential. *Nat Commun* 2025 Feb 16; **16**(1)**:** 1676.

23. Smith T, Felger I, Tanner M, Beck HP. Premunition in Plasmodium falciparum infection: insights from the epidemiology of multiple infections. *Trans R Soc Trop Med Hyg* 1999 Feb; **93 Suppl 1:** 59-64.

24. Nkrumah FK. Changes in the presentation of Burkitt's lymphoma in Ghana over a 15-year period (1969-1982). *IARC Sci Publ* 1984; (63)**:** 665-674.

25. Coghill AE, Proietti C, Liu Z, Krause L, Bethony J, Prokunina-Olsson L*, et al.* The Association between the Comprehensive Epstein-Barr Virus Serologic Profile and Endemic Burkitt Lymphoma. *Cancer Epidemiol Biomarkers Prev* 2020 Jan; **29**(1)**:** 57-62.

26. Boam DS, Clark AR, Docherty K. Positive and negative regulation of the human insulin gene by multiple trans-acting factors. *J Biol Chem* 1990 May 15; **265**(14)**:** 8285-8296.

27. Thomas N, Dreval K, Gerhard DS, Hilton LK, Abramson JS, Ambinder RF*, et al.* Genetic subgroups inform on pathobiology in adult and pediatric Burkitt lymphoma. *Blood* 2023 Feb 23; **141**(8)**:** 904-916.

28. The GTEx Consortium atlas of genetic regulatory effects across human tissues. *Science* 2020 Sep 11; **369**(6509)**:** 1318-1330.

29. Wen J, Sun Q, Huang L, Zhou L, Doyle MF, Ekunwe L*, et al.* Gene expression and splicing QTL analysis of blood cells in African American participants from the Jackson Heart Study. *Genetics* 2024 Jul 26.

30. Cooper EH, Hughes DT, Topping NE. Kinetics and chromosome analyses of tissue culture lines derived from Burkitt lymphomata. *Br J Cancer* 1966 Mar; **20**(1)**:** 102-113.

31. Epstein MA, Barr YM, Achong BG. Studies with Burkitt's lymphoma. *Wistar Inst Symp Monogr* 1965 Sep; **4:** 69-82.

32. de Leeuw CA, Mooij JM, Heskes T, Posthuma D. MAGMA: generalized gene-set analysis of GWAS data. *PLoS Comput Biol* 2015 Apr; **11**(4)**:** e1004219.

33. Maziarz M, Nabalende H, Otim I, Legason ID, Kinyera T, Ogwang MD*, et al.* A cross-sectional study of asymptomatic Plasmodium falciparum infection burden and risk factors in general population children in 12 villages in northern Uganda. *Malar J* 2018 Jun 20; **17**(1)**:** 240.

34. Baik S, Mbaziira M, Williams M, Ogwang MD, Kinyera T, Emmanuel B*, et al.* A case-control study of Burkitt lymphoma in East Africa: are local health facilities an appropriate source of representative controls? *Infect Agent Cancer* 2012 Mar 13; **7**(1)**:** 5.

35. Johnston WT, Mutalima N, Sun D, Emmanuel B, Bhatia K, Aka P*, et al.* Relationship between Plasmodium falciparum malaria prevalence, genetic diversity and endemic Burkitt lymphoma in Malawi. *Sci Rep* 2014; **4:** 3741.

36. Bal E, Kumar R, Hadigol M, Holmes AB, Hilton LK, Loh JW*, et al.* Super-enhancer hypermutation alters oncogene expression in B cell lymphoma. *Nature* 2022 Jul; **607**(7920)**:** 808-815.

37. Kretzmer H, Bernhart SH, Wang W, Haake A, Weniger MA, Bergmann AK*, et al.* DNA methylome analysis in Burkitt and follicular lymphomas identifies differentially methylated regions linked to somatic mutation and transcriptional control. *Nat Genet* 2015 Nov; **47**(11)**:** 1316-1325.

38. López C, Kleinheinz K, Aukema SM, Rohde M, Bernhart SH, Hübschmann D*, et al.* Genomic and transcriptomic changes complement each other in the pathogenesis of sporadic Burkitt lymphoma. *Nat Commun* 2019 Mar 29; **10**(1)**:** 1459.

39. López C, Burkhardt B, Chan JKC, Leoncini L, Mbulaiteye SM, Ogwang MD*, et al.* Burkitt lymphoma. *Nat Rev Dis Primers* 2022 Dec 15; **8**(1)**:** 78.

40. Watier H, Auriault C, Capron A. Does Epstein-Barr virus infection confer selective advantage to malaria-infected children? *Lancet* 1993 Mar 6; **341**(8845)**:** 612-613.

41. Robbiani DF, Deroubaix S, Feldhahn N, Oliveira TY, Callen E, Wang Q*, et al.* Plasmodium Infection Promotes Genomic Instability and AID-Dependent B Cell Lymphoma. *Cell* 2015 Aug 13; **162**(4)**:** 727-737.
